# Complete genome sequence and comparative genomics of the probiotic yeast *Saccharomyces boulardii*

**DOI:** 10.1038/s41598-017-00414-2

**Published:** 2017-03-23

**Authors:** Indu Khatri, Rajul Tomar, K. Ganesan, G. S. Prasad, Srikrishna Subramanian

**Affiliations:** CSIR-Institute of Microbial Technology, Chandigarh, India

## Abstract

The probiotic yeast, *Saccharomyces boulardii* (*Sb*) is known to be effective against many gastrointestinal disorders and antibiotic-associated diarrhea. To understand molecular basis of probiotic-properties ascribed to *Sb* we determined the complete genomes of two strains of *Sb* i.e. Biocodex and unique28 and the draft genomes for three other *Sb* strains that are marketed as probiotics in India. We compared these genomes with 145 strains of *S. cerevisiae* (*Sc*) to understand genome-level similarities and differences between these yeasts. A distinctive feature of *Sb* from other *Sc* is absence of Ty elements Ty1, Ty3, Ty4 and associated LTR. However, we could identify complete Ty2 and Ty5 elements in *Sb*. The genes for hexose transporters *HXT*11 and *HXT*9, and asparagine-utilization are absent in all *Sb* strains. We find differences in repeat periods and copy numbers of repeats in flocculin genes that are likely related to the differential adhesion of *Sb* as compared to *Sc*. Core-proteome based taxonomy places *Sb* strains along with wine strains of *Sc*. We find the introgression of five genes from *Z. bailii* into the chromosome IV of *Sb* and wine strains of *Sc*. Intriguingly, genes involved in conferring known probiotic properties to *Sb* are conserved in most *Sc* strains.

## Introduction

The probiotic yeast *Saccharomyces boulardii* (*Sb*) has unique physiological properties such as tolerance to variations in pH, temperature and local stresses like the presence of GI enzymes, bile salts, and organic acids^1^. Several clinical studies have been performed to present *Sb* as a unique organism that inhibits pathogens^2–5^ and restores gut flora and improves digestion^6, 7^. The mechanism of elimination of the pathogenic bacteria is mainly attributed to the adhesion proteins of *Sb* that binds to bacteria and inhibits their adhesion to the mucous-intestinal membrane^8, 9^. Certain proteins in *Sb* have been reported previously for their key roles in providing protection against *Escherichia coli*, *Clostridium difficile*, *Vibrio cholera*, and *Helicobacter pylori* infections^10^. A 63 kDa protein phosphatase of *Sb* dephosphorylates the *E. coli* endotoxin^11^, a 54 kDa serine protease provides protection against *Clostridium difficile* infections by cleaving toxins A and B^12, 13^ and a 120 kDa heat and trypsin-labile non-proteolytic protein of *Sb* neutralizes the secretions induced by cholera toxin by possibly reducing cyclic Adenosine Monophosphate (cAMP) levels^14^.

A French Scientist Henri Boulard initially isolated *Sb* from the fruits lychee and mangosteen in 1923 and the organism was characterized as ‘*Saccharomyces boulardii*’ a novel species of genus *Saccharomyces* possibly to differentiate its probiotic effects and application from other yeast species^15^. Characterization of *Sb* as a separate species was further supported by the lack of galactose utilization and sporulation as compared to *S. cerevisiae* (*Sc*)^16^. Molecular phylogenetic and typing techniques suggested that *Sb* forms a separate cluster but belong to species *Sc* ^17^. Comparative genomic hybridization experiments also established that *Sc* and *Sb* are different strains of the same species but the loss of all intact Ty1/2 elements was reported only in *Sb*. The loss of Ty elements was hypothesized to be related to *Sb*’*s* non-sporulation and diploidy^18^ as the transcription of these mobile elements is under diploid control^19, 20^. The numbers of Ty elements are maintained via transposition during sporulation and haploid mitotic growth, but the absence of these stages can lead to the loss of Ty elements^21, 22^.

The unique formulations of *Sb*, isolated from different sources like lychee, mangosteen, pineapple, etc., explored for treating different disorders have been patented by different companies and laboratories^23–26^. The mode of action of the probiotic yeast is not completely known^10^, however the beneficial effects of the yeast have been established through various clinical studies^27–33^. However, the probiotic yeast has also been found to be associated with fungemia in immune-compromised patients^34–37^. Our group reported the first genome of *Sb*-*EDRL* strain (Econorm from Dr. Reddy’s Laboratory) and tried to trace the genomic reasons for the probiotic behavior of this yeast^38^. There we explored that the proteins appeared to be specifically present in *Sb* were also present in the *Sc* strains^38^. However, a single draft genome may not be sufficient to study the probiotic properties associated with the organism. Whole genome sequence of more *Sb* strains including complete genomes will be required to understand the evolution and quantitative variations among *Sb* strains.

We have sequenced whole genomes from five *Sb* strains marketed by Laboratories Biocodex, Kirkman Labs, Unisankyo Ltd. (Now Sanzyme Ltd.) and Unique Biotech to find the reasons for probiotic properties of *Sb*. Two strains isolated from *Sb* sachets marketed by Laboratories Biocodex and Unique Biotech was assembled to completion for comparison and to address biologically relevant differences. We report the complete genomes of *Sb*-*biocodex* and *Sb*-*unique28* in this paper along with draft genomes of *Sb*-*kirkman* and *Sb*-*unisankyo* and updated version of the *Sb*-*EDRL* genome. We have analyzed genomes of seven different strains of *Sb* to find if they are different and can account for different probiotic properties or are species specific. We have compared the *Sb*-*biocodex* with *Sb*-*unique28* genome to find differences among these probiotics at the strain level. To address the differences between probiotic yeasts and other strains of *Sc* we compared all five *Sb* genomes with all available genomes of *Sb* and *Sc* strains. This genomic study for probiotic yeast relates to the previous clinical and molecular studies and reports variations at genome level among strains of *Sb* and *Sc*.

## Results

### Complete genomes of *Sb* strains and their characteristics

**Figure 1:**
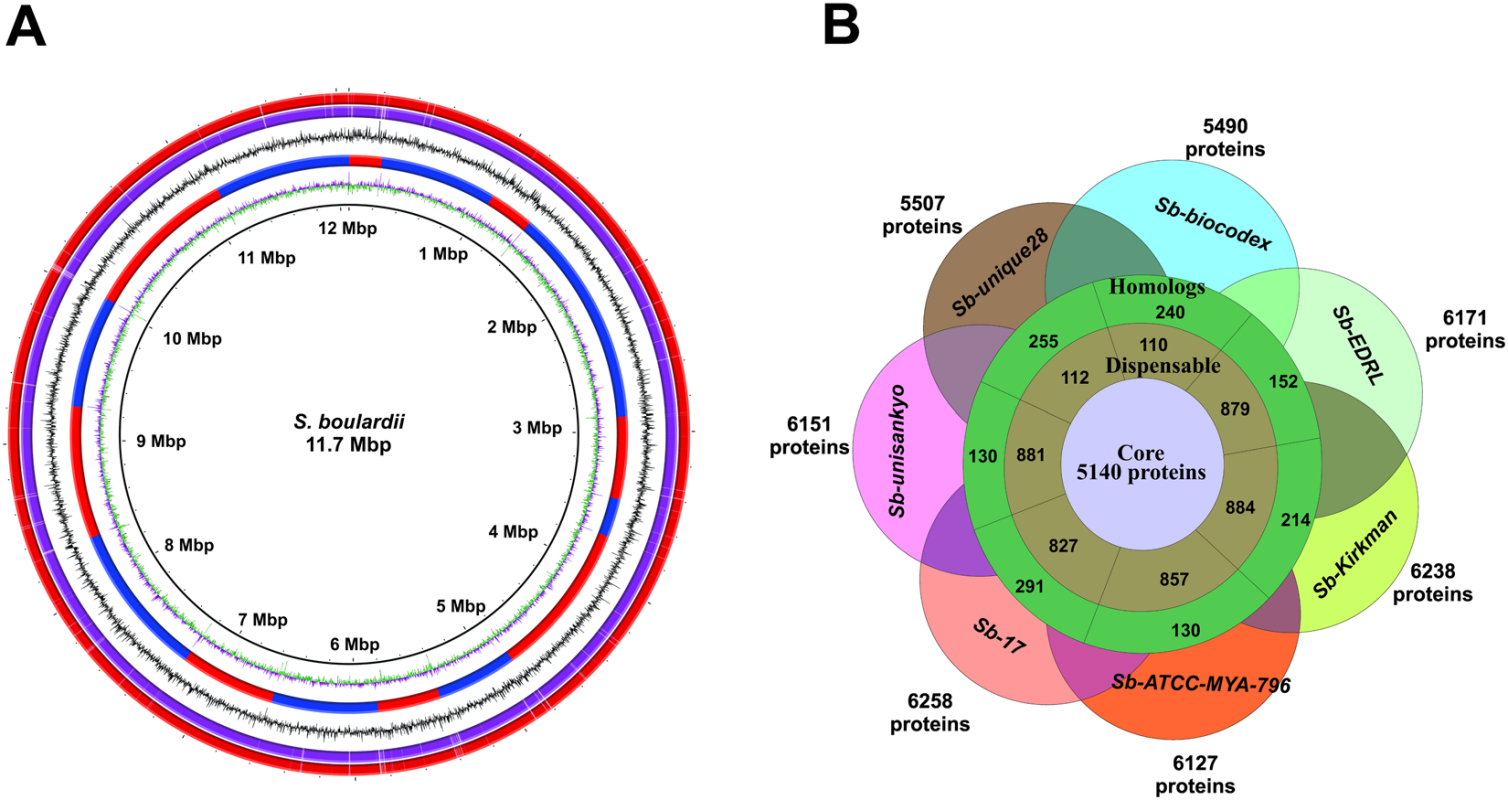
(**A**) Circular representation of the *Sb*-*biocodex* and *Sb*-*unique28* complete genome. Circles (from inside to outside): circle 1 (GC content), circle 2 (*Sc* S288C complete chromosomes with alternative blue and red color); circle 3 (GC skew); circle 4 (*Sb*-*biocodex* complete chromosomes); circle 5 (*Sb*-*unique28* complete chromosomes). BRIG 0.95 was used to build the circular representation. Mapping studies were done using BLASTn with an E-value cut-off 1e-5. (**B**) Core orthologous proteins among all *Sb* proteome represented by orthology diagram depicting dispensable proteome and homologs among these strains.

The complete genomes were obtained for *Sb*-*biocodex* and *Sb*-*unique28* (Fig. 1A) sequenced using PacBio P6C4 chemistry at ~200x coverage. The final assembly of *Sb*-*biocodex* (12 Mbp genome and N50 792,172 bp) comprises of 16 complete chromosomes and 14 unplaced contigs. Similarly, *Sb*-*unique28* was finalized with 14 complete chromosomes, two chromosomes (Chromosome 5 and 9) comprising of two contigs each and nine unplaced contigs (12.1 Mbp genome and N50 929,172 bp) (Table 1; Supplementary File I). Detailed genomic features of *Sb*-*biocodex* and *Sb*-*unique28* are discussed in Supplementary methods (Supplementary Figure 1). The complete chromosomes of *Sb*-*biocodex* and *Sb*-*unique28* were identified with either the telomeres or the telomeric and sub-telomeric regions. The Chromosome I of *Sb*-*biocodex* had a shorter length as compared to *Sb*-*unique28* and *Sc* S288C where we found that the sub-telomeric region of approximately 0.02 Mbp on the right arm with genes annotated as dubious or uncharacterized ORFs (YAR053W to YAR071W) in *Sc* S288C genome (http://www.yeastgenome.org) is absent.

**Table 1:** Genome Assembly statistics of all *Sb* strains.

*Sb* strains	Number of Contigs	N50 (bp)	Genome Size (Mbp)
*Sb*-*biocodex*	31	792,172	12.0
*Sb*-*unique28*	30	909,172	12.1
*Sb*-*EDRL*	77	306,308	11.5
*Sb*-*kirkman*	115	416,209	11.7
*Sb*-*unisankyo*	164	262,146	11.6

*Sb*-*EDRL*, sequenced using 454 sequencing data, was assembled in 107 contigs (Genome Size: 11.5 Mb and N50: 271,789 bp) and was further scaffolded into 77 gapless contigs (Genome Size: 11.5 Mb and N50: 819,652 bp) using Illumina HiSeq PE and MP shotgun data (Table 1). The shotgun reads of *Sb*-*kirkman* and *Sb*-*unisankyo* were assembled in 115 contigs (Genome Size: 11.7 Mb; N50: 621,720 bp) and 164 contigs (Genome Size: 11.6 Mb; N50: 262,146 bp), respectively (Table 1).

Complete 2-micron plasmid was retrieved from *Sb*-*biocodex* and *Sb*-*unique28* matching to *Sc* YJM993 plasmid (Length: 6318 bp; Genbank identifier: CP004528.1). Similarly, the complete circular plasmid was also retrieved from *Sb*-*EDRL*, *Sb*-*kirkman*, and *Sb*-*unisankyo* by mapping their reads to the *Sb*-*biocodex* plasmid sequence. The complete circular plasmid obtained from all the *Sb* strains was found to be 100% identical. G_5297_ -> A and A_5582_ -> G polymorphisms in the *rep2* gene were observed in *Sb* plasmids as compared to *Sc* YJM993 plasmid. The mutation A_5582_- > G was non-synonymous but G_5297_- > A corresponds to A_296_ -> V amino acid change in the Rep2 protein (Supplementary Figure 2).

Approximately 5500 CDS and 300 tRNAs were predicted in all the *Sb* strains (Supplementary File I). The *Sc* S288C genome was also re-annotated using a similar method. All these ORFs were characterized functionally based on the gene names and description provided in Saccharomyces Genome Database (SGD)^39^. The core proteome comprised of 5140 proteins for all *Sb* strains (Fig. 1B) and approximately 200 proteins are found to be unique in each *Sb* strain (Supplementary File II). The unique proteins in all the strains of *Sb* were extracted and subjected to BLASTp against the proteome of all other *Sb* to find if any homologs to those proteins are present in other strains of *Sb*. All these unique proteins have homologs in other strains of *Sb*. All the *Sb* genomes assembled and sequenced in our study were compared with two already available *Sb* genomes (*Sb*-*17* and *Sb*-*ATCC*-*MYA*-*796*) and 145 *Sc* genomes (Supplementary File III).

### Mating Locus and Sporulation in *Sb*

**Figure 2:**
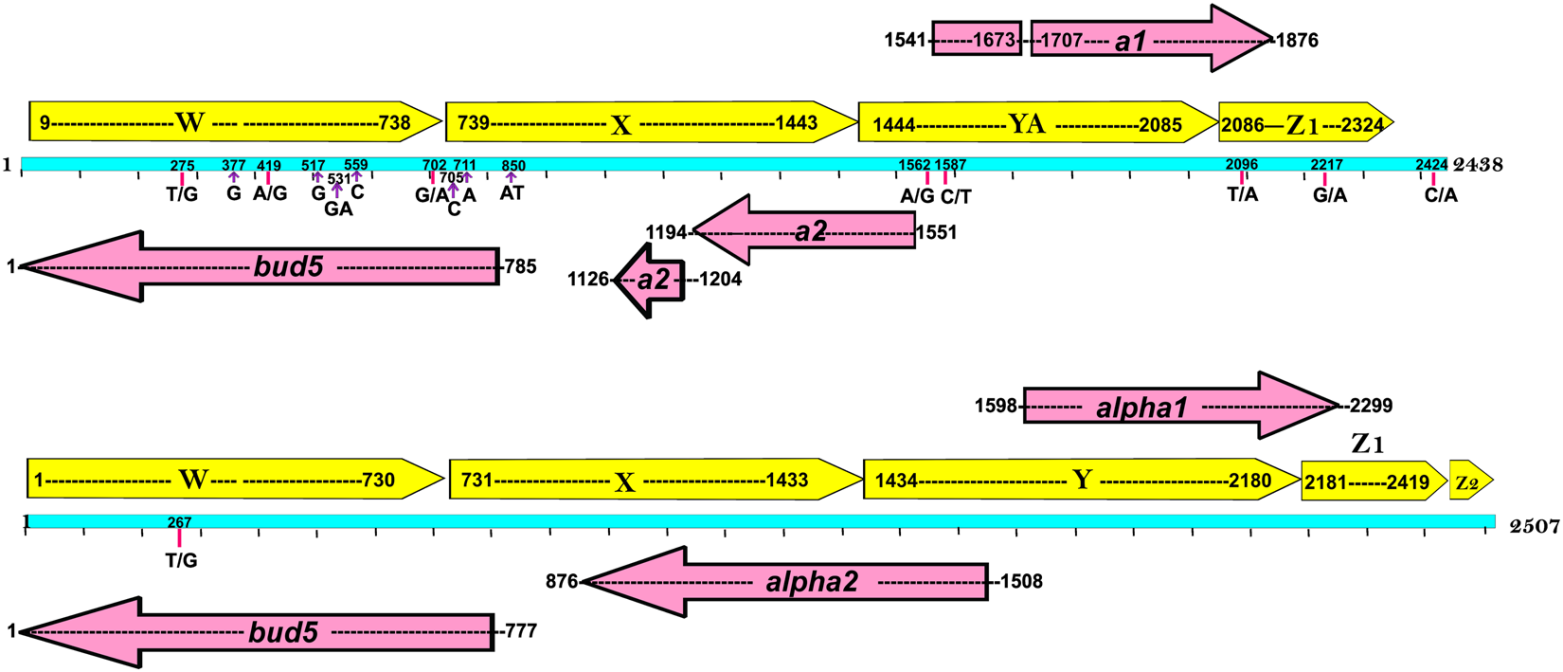
*MAT* Locus of *Sb* biocodex depicting the annotated regions. (**A**) *MATa* locus with yellow arrows depicting the *MAT* region divided in W, X, Y_A_ and Z1 regions with coded *MATa1* and *MATa2* regulatory proteins. (**B**) *MATα* locus with yellow arrows depicting the *MAT* region divided in W, X, Y, Z1 and Z2 regions with coded *MATα1* and *MATα2* regulatory proteins.

Mating type of yeast was determined by the two different alleles of Mating-type (*MAT*) Locus *MATa* and *MATα* ^40^. *Sb* has been suggested to be diploid in previous studies^1, 41, 42^ and should comprise both *MATa* and *MATα* sequences on the Chromosome III at a heterozygous locus. The characterized locus in *Sc* was used as a query to search the *Sb*-*biocodex* genome and both *MATa*, and *MATα* sequences were retrieved from *Sb*-*biocodex* at 99% identity in chromosome III and unplaced scaffold, respectively. *MATa* sequence in *Sb*-*biocodex* is 2438 bp in length which is 99.7% identical to *Sc MATa* locus (GI: V01313) with eight substitutions and seven insertions (Fig. 2). The region was divided into W, X, YA and Z1 regions based on the alignment to *MATa* locus of *Sc* (GI: V01313). Similarly, the *MATα* gene was retrieved by subjecting the *MATα* region of *Sc* S288C from SGD to BLASTn against *Sb* genomes. The *MATα* gene was 2507 bp with only one substitution T_267_ -> G and could be divided into W, X, Y, Z1 and Z2 regions based on the alignment to *Sc* S288C *MATα* gene. The ORFs coded by *MATa* and *MATα* were identified through Augustus, and very short ORFs were predicted using DNA to protein translation tool (http://insilico.ehu.es/translate/). In yeast the *MATα* locus codes for *bud5*, *MATα1*, and *MATα2* genes and *MATa* locus codes for *bud5*, *MATa1*, and *MATa2* genes. Homothallic switching endonuclease (*HO*) gene is required for gene conversion at MAT Locus in haploid cells^43^. In heterothallic strains T189A, G223S, L405S, H475L substitutions and deletion of 36 amino acids (524–559) in HO protein results in loss of endonuclease activity^44, 45^ whereas homothallic cells express *HO* ^45^. The *HO* gene in all strains of *Sb* did not have any of the above-mentioned substitutions or deletions, and hence, the probiotic yeast *Sb* is homothallic diploid wherein both the *MAT* loci are present in the genome.

As it is known that both the heterothallic and homothallic diploid strains sporulate under conditions of nutrient deficiency^46^, we performed sporulation phenotype assay (detailed in Supplementary Methods), to investigate the sporulation in *Sb*. We found that the *Sb* did not sporulate on sporulation media even after one week of incubation concordant with previous studies stating non-sporulation behavior of the probiotic yeast^41^. To investigate the role of sporulation proteins in the non-sporulating behavior of *Sb* the presence and the absence of all the sporulation proteins mentioned in SGD were identified using BLASTp in *Sb* proteome. Further, to rule out the possibility of the lack of any meiotic and mitotic genes hindering the sporulation pathway, we searched for 110 meiotic genes and 56 mitotic genes in *Sb* genome and found all were present (Supplementary File IV). An earlier report suggesting the divergence in CDC16, DMC1, and MND2 sequences as a possible reason for the defective sporulation was also investigated^41^; but all these proteins of *Sb* were >99% identical to the respective proteins in *Sc*. Also, *Sb* was grown on non-fermentable carbon source (Glycerol) to find if the non-sporulation of *Sb* is governed by respiration-sensing pathway^47^ (detailed in Supplementary Methods). The growth of *Sb* was found on the non-fermentable carbon source (Supplementary Figure 3). Thus, the non-sporulation phenotype of *Sb* is unlikely be due to a mutation in sporulation and respiration pathway genes. Since functional *MAT* loci are also critical for sporulation^48^, we speculate that mutations at these loci, as seen in *MATa* sequence in *Sb*-*biocodex* with eight substitutions and seven insertions compared to *Sc MATa* (Fig. 2), might be responsible for the non-sporulation phenotype of *Sb*.

### Gene copy number variations in *Sb* strains as compared to *Sc*

#### Genes absent

The mapping studies to *Sc* genes of yeastmine database^49^ revealed that 50 genes had no reads mapped onto them. Also, we found that 94 genes apart from these 50 genes had read coverage <20 reads. The absence of these genes was confirmed by subjecting these genes as a query to BLASTn against the *Sb*-*biocodex* and *Sb*-*unique28* PacBio assembly with complete chromosomes. Out of these 144 genes, 85 were dubious ORFs, 32 were uncharacterized genes, and 27 were functionally verified genes. The verified genes include two maltase utilization genes (*MAL11* and *MAL13*), two hexose transporters (*HXT9* and *HXT11*), four asparagine catabolism gene (*ASP3*-1, *ASP3*-2, *ASP3*-3 and *ASP4*-4), three palatinose utilization genes (*IMA2*, *IMA3*, *IMA4*), two putative membrane glycoprotein (*VTH1* and *VTH2*) and *ARN2*, *REE1*, *AYT1*, *AIF1*, *COS10*, *ENB1* and *BDS1* (Table 2). Except *ASP3* locus, all these absent genes belong to telomeric or subtelomeric regions of chromosome.

**Table 2:** Absent genes in *Sb*-*biocodex* and *Sb*-*unique28*.

Systematic	Genes	Gene Function
YOL165C	*AAD15*	Aryl-Alcohol Dehydrogenase
YNR074C	*AIF1*	Mitochondrial cell death effector
YHL047C	*ARN2*	Transporter
YLR155C	*ASP3*-*1*	Cell-wall L-asparaginase II involved in asparagine catabolism
YLR157C	*ASP3*-*2*	Cell-wall L-asparaginase II involved in asparagine catabolism
YLR158C	*ASP3*-*3*	Cell-wall L-asparaginase II involved in asparagine catabolism
YLR160C	*ASP3*-*4*	Cell-wall L-asparaginase II involved in asparagine catabolism
YLL063C	*AYT1*	Acetyltransferase
YOL164W	*BDS1*	Bacterially-derived sulfatase
YLR465C	*BSC3*	Bypass of Stop Codon
YNR075W	*COS10*	Protein of unknown function
YGR295C	*COS6*	Protein of unknown function
YOL158C	*ENB1*	Endosomal ferric enterobactin transporter
YOL156W	*HXT11*	Putative hexose transporter that is nearly identical to Hxt9p
YJL219W	*HXT9*	Putative hexose transporter that is nearly identical to Hxt11p
YOL157C	*IMA2*	Isomaltase
YIL172C	*IMA3*	Isomaltase
YJL221C	*IMA4*	Isomaltase
YGR289C	*MAL11*	High-affinity maltose transporter (alpha-glucoside transporter)
YGR288W	*MAL13*	MAL-activator protein
YIR041W	*PAU15*	seripauperin
YKL224C	*PAU16*	seripauperin
YJL217W	*REE1*	Cytoplasmic protein involved in the regulation of enolase (ENO1)
YAL064C-A	*TDA8*	Topoisomerase I Damage Affected
YOR068C	*VAM10*	Vacuolar Morphogenesis
YIL173W	*VTH1*	Putative membrane glycoprotein
YJL222W	*VTH2*	Putative membrane glycoprotein

Also, the yeastmine database genes were subjected to BLASTn against all strains of *Sc* included in this study to find genes unique to *Sb* (Supplementary File V). *MAL11*, *MAL13*, *and ARN2* were present in more than 70% of the strains of different subgroups of *Sc* strains but were absent in all the probiotic strains. We found that the *BDS1* gene was present in tree isolates, laboratory strains, and environmental samples but was absent in wine strains, beer strains, clinical, fruit derived, bakery and bioethanol producing strains. Similarly, the *REE1* gene was present in tree isolates, laboratory strains and environmental samples and a few strains belonging to subgroup wine, beer and clinical but was absent in probiotic *Sb* strains.

The *ASP3* locus was present in more than 80% of the laboratory or industrial strains and bioethanol producing strains and *Sc* strainYJM1383, a fruit derived strain; *Sc* strain CLIB324, a bakery strain, and a few clinical strains (YJM248, YJM339, YJM451, YJM693, YJM1078, and YJM1311). It was absent in the wine, distillery, and probiotic strains. The ORFs coding for this locus on chromosome XII is adjacent to the ribosomal DNA locus. The hexose transporter family is large and comprises of *HXT1*-*17* genes^50^ of which *HXT11* and *HXT9* were absent from all strains of *Sb*. *HXT11* and/or *HXT9* null mutants of *Sc* are resistant to cycloheximide, sulfomethuron methyl, and 4-NQO (4-nitroquinoline-N-oxide)^51^ indicating *Sb* strains also could be resistant to these chemicals.

#### Multi-copy genes

The variation in the copy number of genes in a genome can have phenotypic and physiological differences^52^. Genes for PAU proteins, a member of the seripauperin multigene family, were found to be present in 18–20 copies in the genome, and *gag*-*pol fusion* genes were present in 15 copies in the whole genomes of *Sb*-*biocodex* and *Sb*-*unique28* (Table 3). *THI13* is present in five copies at the sub-telomeric regions and *IMD3* and *COS3* are present in four copies at the telomeric regions of *Sb* chromosomes. Imd3 catalyzes the rate-limiting step in the *de novo* synthesis of GTP^53^ and Cos3 is involved in salt resistance^54^ in *Sc*. The clusters of duplicated and triplicated genes mostly encode stress-related proteins, elongation factors, ribosomal proteins, kinases and transporters, fluoride export and altering replication stress tolerance. These duplicated genes could be helping in better adaptation of *Sb* to the harsh conditions of the mammalian host.

**Table 3:** Multicopy genes in *Sb*-*biocodex*, *Sb*-*unique28 and Sc S288C*.

	*Sb biocodex*	*Sb unique28*	*Sc S288C*
Total clusters	163	150	146
Clusters with two proteins	148	137	127
**Clusters with more than two proteins**			
**Genes in multiple copies**	***Sb biocodex***	***Sb unique28***	***Sc S288C***
Seripauperin PAU	18	20	7
gag-pol fusion proteins	16	16	49
Thi13	5	6	1
IMP dehydrogenase IMD3	4	2	1
Cos3p	4	5	1
YIL169C-like protein	3	4	2
Aad4p	3	2	1
Fex1p	3	3	1
ribosomal 60S subunit protein L2B	3	4	1
Hsp32p	3	3	1
Y’ element ATP-dependent helicase protein 1 copy 1	2	5	5

### Ty elements in *Sb*

Ty1, Ty3, and Ty4 elements were absent in all the *Sb* genomes whereas Ty2 elements were present in *Sb*-*biocodex*, *Sb*-*kirkman* and *Sb*-*unique28* and one Ty5 element was present in all *Sb* strains except *Sb*-*17* and *Sb*-*MYA*-*796* (Supplementary Figure 4). Also, the presence of genes encoding *gag*-*pol* and *gag*-*co*-*pol* fusion proteins was confirmed by read mapping and coverage analysis. These were either contained in the Ty2 or Ty5 elements. The presence of Ty elements was compared in between complete genomes of *Sb viz. Sb*-*biocodex* and *Sb*-*unique28*; where the elements are present in the same chromosomes with some deviations in positions (Table 4; Supplementary File VI). Ty1–4 elements integrate near tRNA or RNA polymerase III genes^55^, but analyzing the neighboring genes of these Ty elements, we found only one Ty element in each of *Sb*-*unique28* and *Sb*-*biocodex* has Ribosomal 40S subunit protein upstream of it.

**Table 4:** Distribution of Ty elements in *Sb* strains.

	*Sb-17*	*Sb-biocodex*	*Sb-EDRL*	*Sb-kirkman*	*Sb-MYA-796*	*Sb-unique28*	*Sb-unisankyo*	*Sc* S288C
gag	1			1	1	1	1	1
gag co pol		3	5	3		3	1	45
gag pol		2				3		45
pseudo	2		2	2	2	2	2	2
Suppressor SPT7	1	1	1	1	1	1	1	1
Ty1 element								31
Ty1 LTR	147	191	173	190	151	200	169	483
Ty2 element		10		1		7		13
Ty2 LTR	5	4	12	9	8	14	11	32
Ty3 element								2
Ty3 LTR	26	46	40	45	29	45	33	42
Ty4 element								3
Ty4 LTR	15	16	17	17	15	17	15	35
Ty5 element		1	1	1		1	1	1
Ty5 LTR	5	3	3	4	4	3	3	8
Ty A								1
Ty B								1

### Flocculation and adhesion

*FLO1*, *FLO5*, *FLO8*, *FLO9*, *FLO10*, *FLO11*, *FIG2*, and *AGA1* encode flocculation proteins that belong to yeast adhesin families, and their sufficient expression leads to flocs, flor, biofilms or filaments formation by either binding to other yeast cell receptors or foreign surfaces^56^. The protein sequences of these flocculins obtained from SGD were subjected to BLASTp against *Sb* and *Sc* proteomes (Supplementary File VII). Flocculation genes are characterized by the presence of a large number of repetitive sequences with linear correlation to their size^57^. *FLO1* and *FLO5* are paralogs that arose from segmental duplication^58^ of which we could trace the presence of *FLO1* gene in our *Sb* genomes. *FLO8*, *FLO10*, *FLO11*, *FIG2*, and *AGA1* were also present in all strains of *Sb*. *FLO1*, *FLO5*, *FLO8*, *FLO9*, *FLO10*, and *FLO11* are telomeric genes and have repeats^59, 60^. These were searched in other *Sc* genomes, but we found that most of them encode truncated proteins possibly owing to limitations of sequencing technology. In *Sb* genomes, we found that these genes encode either truncated proteins or could not be traced in *Sb*-*EDRL*, *Sb*-*kirkman*, *Sb*-*unisankyo*, *Sb*-*17*, and *Sb*-*MYA*-*796*. The complete sequences of flocculins from *Sc* and *Sb* strains were retrieved and were aligned using MUSCLE^61^ and were edited manually to trace variation in the number and period of the repeats in sequences (Supplementary Figure 5). Seven copies of 45 residues were identified in FLO1 protein of *Sb* and *Sc* YJM1385 (fruit borne) and *Sc* YJM1129 (brewery strain) whereas only one copy of this period was present in other *Sc* strains. The FLO8 protein of *Sb* was identical to that in most of the *Sc* strains except *Sc* strains S288C, BY4741, BY4742, FY1679, JK9-3d, SEY6210, W303, X2180-1A and YPH499 where a point mutation has resulted in the truncation of the protein^62^. FLO10 and FLO11 domains are present in all strains of *Sb* with repeats where the copy number and a period length of the repeats were similar to all the *Sc* strains.

### Introgression of *Zygosaccharomyces bailii* proteins into *Sb* and *Sc* wine strains

**Figure 3:**
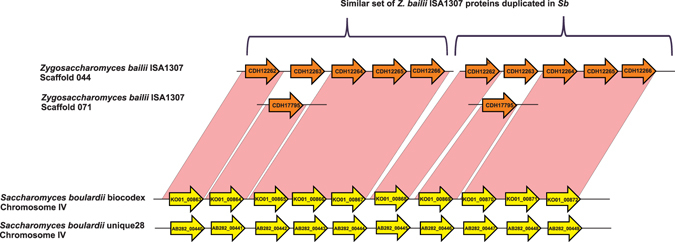
Introgression of five genes in two copies from *Zygosaccharomyces bailii* to Chromosome IV of *Sb* genomes.

Ten genes on chromosome IV of *Sb*-*biocodex* and *Sb*-*unique28* were found to encode proteins >90% identical to *Zygosaccharomyces bailii* ISA1307 proteins (Fig. 3). On investigating this region, we found that five genes of *Z. bailli* had introgressed and further undergone duplication. Three of these genes were annotated as encoding uncharacterized proteins; one encodes a probable 5-oxoprolinase, and one is an allantoate transporter. These genes were searched in the sequenced *Sc* strains too, where a single copy of these genes was present in similar fashion in *Sc* UFMG A-905 strain with probiotic properties and wine strains YJM339, RM11-1a, L1528, and YS9. Three copies of these genes were also present in *Sc* wine strain BC187. Few of these introgressed genes were also present in bioethanol producing strain *Sc* JAY291; wine strains *Sc* EC1118, Vin13, VL3, AWRI796 in single copy and *Sc* wine strain LalvinQA23 has two copies of these *Z. bailli* genes.

### Taxonomic position of *Sb*

**Figure 4:**
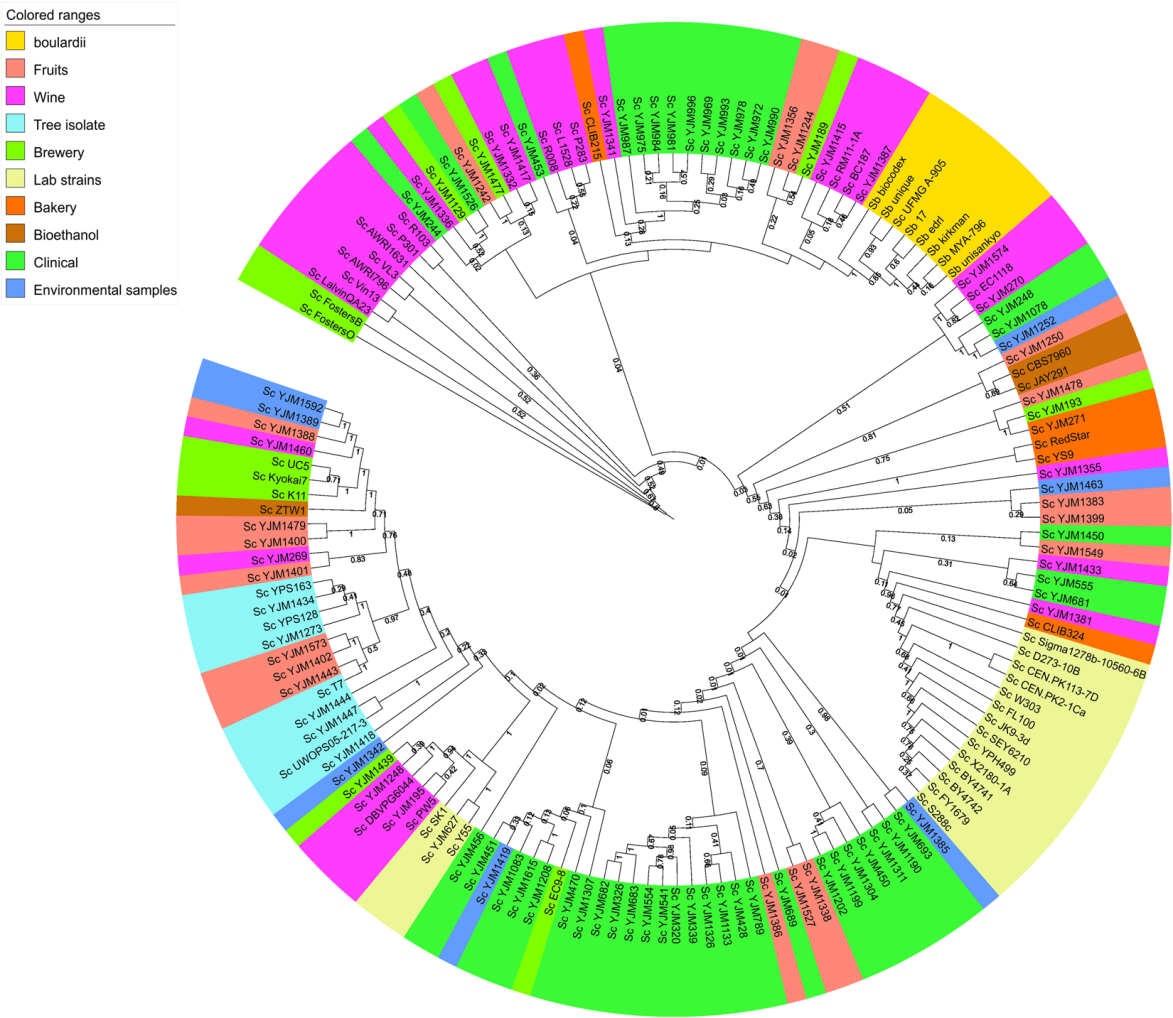
Maximum Evolution tree for 182 orthologous proteins of all strains of *Sb*, *Sc* and *S. kudriavzevii* as outgroup species. The taxa are shaded based on the isolation source of strains as boulardii: gold; Fruits: lightsalmon; Wine: hotpink; Tree isolate: skyblue; Brewery: lawngreen; Lab strains: palegoldenrod; Bakery: darkorange; Bioethanol: peru; Clinical: mediumseagreen; Environmental samples: cornflowerblue.

*Sb* and *Sc* shares more than 99% genomic relatedness as determined by Average Nucleotide identity (ANI) (Supplementary File VIII). Thus we retrieved the core proteome from all 145 *Sc* strains and 7 *Sb* strains and a outgroup species *S. kudriavzevii*. 182 proteins for which orthologs could be obtained in all 153 organisms were retreived and concatenated to find the taxonomic position of *Sb* with comparison to *Sc*. The ML based tree rooted the tree at *S. kudriavzevii*, the outgroup species, which further clustered all the *Sc* strains (Fig. 4). The *Sc* strains in the phylogenetic tree were grouped as per their isolation source. All the *Sb* strains were grouped in a clade, where *Sc* UFMG A-905 strain is closer to *Sb*-*unique28*. *Sb*-*biocodex* was present at the root of the *Sb* clade that groups *Sb*-*17*, *Sb*-*EDRL*, *Sb*-*unisankyo*, *Sb*-*kirkman*, and *Sb*-*MYA*-*796*. The *Sb* strains share the clade with wine strains *Sc* strains BC187, YJM1387, YJM1417, YJM1332 and R008, brewery strains *Sc* YJM1477 and *Sc* strain YJM1242 isolated from fruits. Separate clusters were observed in case of laboratory strains whereas clinical isolates were grouped into three distinct clusters in the tree. *Sc* strains isolated from fruits were scattered across tree but were closer to *Sc* wine strains or tree isolates. In terms of taxonomy, it is clear that the *Sb* strains are closely related to the *Sc* wine strains, and it would be of interest to explore the probiotic potential of these wine strains.

## Discussion

The complete genomes of *Sb* determined in this study are the best assemblies of the yeast known, as the long PacBio reads used in the study assisted in the identification of complete chromosomes, telomeres and complete structures of Ty elements which could not be identified using Illumina HiSeq short read data. The comparative genomic hybridization analysis for *Sb* revealed at the first place that the Ty1-2 elements were absent from *Sb* ^18, 41^ which could not be identified in *Sb*-*EDRL* and *Sb*-*unisankyo* draft assemblies but in complete genomes of *Sb*-*biocodex* and *Sb*-*unique*-28, we were able to identify 15 complete Ty elements. Chromosome IX has been mentioned to exhibit trisomy^1^, but such event could not be traced instead we found that the chromosome XII had double the read coverage (400x) as compared to the other chromosomes (200–270x) which could be owing to the aneuploidy of the chromosome XII. The *rep2* gene of 2-micron circle plasmid had G_5297_ -> A synonymous mutation resulting in A_296_ -> V amino acid change as reported earlier^63^. *Sb* strains share ~3100 orthologous proteins with one or more *Sc* strains suggesting large conserved protein repertoire between *Sc* and *Sb*. The *Sb* proteome had high conservation level among the strains of *Sb* owing to the presence of the protein homologs in one or the other strains of *Sb*. *Sb* strains are likely to be homothallic and diploid due to the presence of both *MAT* alleles and *HO* gene. Being diploid the yeast should sporulate, but the sporulation was absent in *Sb* as revealed by sporulation assay which has already been established in a previous study^1^. The respiration efficiency of *Sb* strains was determined by its growth on non-fermentable carbon sources^47^ and hence the non-sporulation behavior of the organism is not owing to the respiration deficiency. Even the absence or divergence of any sporulation gene was not observed in *Sb* proteome in comparison to that of *Sc*. Possibly the deficiency of the mating type genes^48^ in *Sb* could be leading to the non-sporulation behavior.

*Sb* can prevent antibiotic-associated diarrhea, recurrent *Clostridium difficile*-associated diarrhea and colitis, Traveller’s diarrhea, acute bacterial and viral diarrhea, anti-inflammatory bowel diseases by various mechanisms^10^. *Sb*, with antimicrobial properties, secretes 54 kDa^12, 13^, 63 kDa^11^, and 120 kDa^64^ proteins that exhibit the protection of gut microflora against pathogenic bacteria either through cleaving the toxin or by reducing the cAMP level. These proteins were searched in *Sb* and *Sc*, and it was established that these proteins are not unique to *Sb* but were present in *Sc* too^38^ (Supplementary Methods). *Sb* has been shown to hinder the biofilm formation by pathogenic strains because of steric hindrance caused by its larger size as compared to bacteria^65^. Also, *Sb* exerts its antimicrobial effect by adhering to intestinal mucus membrane and eliminating pathogens by flow preventing their adhesion to the intestine^8^. Adhesion to other foreign surfaces has been reported to be a critical step for pathogenic as well as a probiotic organism^57^. Yeast also chooses its lifestyle according to its environment and can form different colonies such as non-adhesive colonies, self-adhesive non-dissolvable colonies, biofilms during foreign adhesion or flocs or flor^56^. All flocculin genes harbor a large number of repeats that tend to increase or decrease in copy numbers affecting the degree of flocculation and sensitivity to stress conditions^56^. We could identify complete flocculin genes in the whole genomes of *Sb* where the repeats and their copies were varying even within *Sb* genomes but were consistent in between *Sb*-*biocodex* and *Sb*-*unique28*. *Sb* harbors all the flocculin genes required for protection against environmental stress as ethanol and fungicides (*FLO1*)^66^, floc formation (*FLO10* and *FLO11*)^67^, and biofilm formation (*FLO10*, *FLO11*, and *FIG2*)^67, 68^. *FLO8* gene required for expression of *FLO1* and *FLO11* was present in *Sb*, but was truncated in laboratory strains, impairing their flocculation and adhesion to the foreign surfaces^62^. These genes are located at telomeres and are highly repetitive, and the maximum number of repeats identified in the strains of *Sb* could be conferring higher adhesive properties to the organism.

During evolution, the *Saccharomyces* yeast can undergo a process of gene duplication, polyploidy, chromosomal rearrangements, interspecific hybridization, and introgression^69^. The process of eukaryote-to-eukaryote gene transfer events and introgression in *Sc* strains have been validated through genetic experiments and certain regions among *Sc* strains have been reported to have similarity with *Z. bailii*, *S. arboricola*, *S. bayanus* and other yeasts^70, 71^. A particular region in wine yeast *Sc* EC1118 has been proposed earlier to be transferred from *Z. bailii* type strain CBS680^70^. Similarly, a specific region of 10 proteins in chromosome IV of *Sb* was syntenic to the five proteins in *Z. bailii* ISA1307 which has got duplicated in *Sb* and few wine strains of *Sc*.

The taxonomic position of *Sb* as a separate species has been controversial^10, 41, 72–75^. It was initially considered as a separate species of genus *Saccharomyces*, but CGH analysis characterized it as a strain of *Sc*. The core proteome based phylogeny, obtained from the *Sb*, *Sc*, and *S. kudriavzevii* could resolve the clades. All *Sb* strains got clustered together in a clade along with *Sc* UFMG A-905 strain, a *Sc* strain with probiotic properties where *Sb*-*unique28* shared the sister clade with *Sc* UFMG A-905. All the phylogenies drawn revealed concordantly that the wine strains of *Sc* are closer to the *Sb*.

There is no doubt that the *Sb* belongs to *Sc* species and is a strain of *Sc*, but the probiotic features of *Sb* make it a yeast with beneficial effects in gastrointestinal disorders^3, 5, 7, 12, 14, 24, 26, 41, 76–79^. The genomic perspective of the organism with relevance to its probiotic features was examined in the study where we couldn’t identify any specific and unique proteins in *Sb*, since the *Sb* proteins are homologous with one or the other proteins of *Sc* strains. The genomic perspective in this study revealed that the *Sb* probiotic strains are closer to wine strains of *Sc* than industrial or baking strains; as revealed by the absence of *ASP3* locus, the introgression of *Z. bailli* proteins and the core proteome based taxonomic placement of probiotic *Sb* and wine *Sc* strains. *Sc* BC187 a wine strain shows maximum similarity with the *Sb* strains and might be explored for its probiotic properties similar to *Sc* strain UFMG A-905^80, 81^.

## Methods

### Isolation and purification of *Sb* genomic DNA

The lyophilized yeast *Sb* available in the market as probiotics in sachets were used for isolation of source DNA for *Sb*-*biocodex* (Florastor), *Sb*-*EDRL* (Dr. Reddy’s Laboratories) and *Sb*-*unique28* (Unique Biotech). Two cultures of *Sb* from Unisankyo Ltd. (Now Sanzyme Ltd.) and Kirkman Labs that were maintained at MTCC (IMTECH, India) since 2003 were obtained for DNA isolation and sequencing. DNA isolation was performed using the ZR Fungal/Bacterial DNA miniprep kit (Zymogen), and purity index was checked as the ratio of OD at 260/280 nm was >1.8 as observed by NanoDropND-1000 spectrophotometer. Detailed isolation and purification methods have been provided in supplementary material.

### Genome Sequencing

*Sb*-*unique28* and *Sb*-*biocodex* were sequenced using PacBio P6C4 chemistry using eight and nine SMRT cells, respectively. 101-bp paired-end (PE) shotgun data from Illumina HiSeq-1000 high-throughput sequencing technology was also obtained for *Sb*-*biocodex*. The sequencing depth for PacBio sequencing was approximately 200x for both *Sb*-*biocodex* and *Sb*-*unique28*. *Sb*-*EDRL* was sequenced earlier using Roche 454 sequencing technology which now has also been sequenced on Illumina HiSeq 1000 platform to obtain 101-bp PE shotgun data twice and one 2 K and one 8 K mate-pair (MP) library shotgun data. *Sb*-*kirkman* was sequenced using Illumina HiSeq1000 to get 101-bp PE shotgun data along with 2 K and 8 K MP reads. For *Sb*-*unisankyo*, only 101 bp shotgun data was obtained from Illumina HiSeq 1000 sequencing platforms. The Illumina HiSeq-1000 sequencing was performed at C-CAMP, Bangalore, India and PacBio P6C4 chemistry SMRT cells sequencing data was obtained from Genome Quebec Centre, McGill University, Canada. Detailed Sequencing protocols have been provided in supplementary material.

### Genome Assembly and Annotation

*Sb*-*biocodex* and *Sb*-*unique28* SMRT cells were assembled using Hierarchical Genome Assembly Process (HGAP) v2.0^82^ pipeline of the SMRT Portal. The *Sb*-*biocodex* and *Sb*-*unique28* contigs were aligned to *Sc* reference strain S288C using Mauve aligner^83^ and the contigs completely mapping to the chromosomes were submitted to NCBI as complete chromosomes. Some unplaced contigs were obtained which were subjected to BLASTn^84^ against the finalized chromosomes of respective strains and the NT database to find if any contig belongs to a plasmid or mitochondrial genomes.

*Sb*-*EDRL*, assembled earlier with Roche 454 shotgun data and submitted to NCBI (ATCS01000000), was further scaffolded and gapfilled using SSPACE v3.1^85^ and GapFiller v1.10^86^ with Illumina PE and MP data. *Sb*-*kirkman* and *Sb*-*unisankyo* were assembled *de novo* using SPAdes v3.1^87^. Further, these assemblies were scaffolded using SSPACE v3.0 and gapfilled using GapFiller v1.10. Also, the Illumina Next-Generation Sequencing (NGS) data for four *Sb*: *Sb*-*biocodex*, *Sb*-*EDRL*, *Sb*-*kirkman* and *Sb*-*unisankyo* were mapped on to the 16 chromosomes of *Sb*-*biocodex* to find the gaps that were not covered by Illumina reads. Final assemblies were submitted to GenBank under accession numbers *Sb*-*biocodex*: LIIL01, *Sb*-*unique28*: LIOO01, *Sb*-*kirkman*: LOMX01, *Sb*-*unisankyo*: LNQF01 and *Sb*-*EDRL* ATCS02.

All the *Sb* strains were annotated using Augustus^88^ as gene predictor with species model ‘*Sc* S288C’ and tRNA was predicted by tRNAscan-SE 1.23^89^. Features thus annotated were subjected to BLASTp against Saccharomyces Genome Database (SGD)^39^ protein dataset and non-redundant (NR) protein sequence database for functional characterization of the proteins with an E-value cutoff of 1e^−5^.

### Gene copy number variations

The complete set of *Sc* genes present in yeastmine database^49^ were mapped by the Illumina shotgun reads of *Sb* strains *Sb*-*biocodex*, *Sb*-*kirkman*, *Sb*-*unisankyo*, and *Sb*-*EDRL*. Genes with no read coverage were checked in the complete PacBio genome assembly of *Sb*-*biocodex* for their absence. The genes duplicated within *Sb*-*biocodex* and *Sb*-*unique28* genomes with >90% identity, and >90% query coverage were identified.

### Presence-Absence Variations (PAVs)

The proteins or genes involved in adhesion, flocculins, sporulation, meiotic, mitotic, galactose utilization and palatinose utilization were downloaded from SGD and were subjected to BLASTp or BLASTx against the proteome of all *Sb* and *Sc* and the hits thus obtained were filtered at >50% query coverage and >30% identity. The duplicate hits were removed, and the PAVs were plotted as matrix across all 7 *Sb* and 145 *Sc* genomes.

### Genome datasets used for comparison

The *Sb* genomes were compared to 145 strains of *Sc* reported at SGD and NCBI. The annotations for all strains of *Sc* were obtained from SGD and NCBI. NCBI reports 168 genomes as on 01-May-2015 of which 50 were reported in SGD. Five strains (*Sc* FL100, *Sc* RM11-1a, *Sc* Sigma1278b, *Sc* W303 and *Sc* YPS163) were excluded as their updated versions were considered for the analysis. Two strains (*Sc* CLIB382 and *Sc* M22) were excluded from annotations as the number of scaffolds was more than 6000. *Sc* strain T73 and Y10 were also eliminated as the number of annotated features was less than 3000. Fourteen strains with no annotations available at NCBI and SGD were not used for the comparative analysis. Unannotated *Sc* strain UFMG A-905 available from NCBI was utilized in the comparative study as the strain has been mentioned to exhibit probiotic effects. Two *Sb*-*17* and *Sb*-*ATCC*-*MYA*-*796* were also used for the comparison. The features prediction was made using Augustus^88^ for these strains. All these strains were divided into nine subgroups based on the origin of the strains as Fruit-derived, Wine, Tree-isolates, Beer, Laboratory, Bioethanol, Bakery, Clinical and Environmental.

### Identification of Ty elements

Retrotransposons sequences downloaded from SGD database were subjected to BLASTn against the *Sb* genomes. The BLAST results obtained were further filtered with query coverage of 90% and best hits were retrieved. Further, the matched regions were screened manually.

### Core-proteome Analysis

The orthologous pairs of proteins across all *Sb* and *Sc* proteomes were extracted using Proteinortho v2.3^90^ PERL script and was considered as the core proteome of all *Sb* strains. The homologs of proteins of one *Sb* strain were identified by subjecting the proteins to BLASTp against proteome of other *Sb* strains.

### Taxonomy

The core proteins retrieved from proteome of *Sb* and *Sc* and outgroup species *S. kudriavzevii* IFO 1802 were concatenated and aligned using MAFFT^91^. The alignment was fed to MEGA v6.0^92^ for generation of Minimum Evolution (ME) tree at 100 bootstrap values. The evolutionary distances were calculated using Dayhoff matrix based method.

### Data access

This Whole Genome Shotgun project has been deposited at DDBJ/EMBL/GenBank along with the plasmid and ITS sequences retrieved from all *Sb*. The version described in this paper is the first version for *Sb*-*biocodex*- LIIL01000000, *Sb*-*unique28*- LIOO01000000, *Sb*-*kirkman*- LOMX01000000, *Sb*-*unisankyo*- LNQF01000000 and second version of *Sb*-EDRL- ATCS02000000.

## Conclusions

Two complete genomes and three draft genomes of *Sb* were sequenced and assembled. The complete genomes revealed the presence of Ty2 elements and gag-co-pol genes in *Sb* unlike the complete absence of Ty1/2 elements in *Sb* suggested previously. Homozygous diploid probiotic yeast *Sb* had non-sporulation phenotype for which the absence/divergence of sporulation genes or respiration efficiency is not responsible, but the deficiency in mating genes may be playing a role.

The physiological and molecular differences making *Sb* different from *Sc* were explored through genome analysis. We found that the *HXT11* and *HXT9* hexose transporter genes were absent in *Sb* only but were present in all *Sc* strains. The asparagine utilization (*ASP3*) locus was absent in *Sb* and *Sc* wine and distillery strains and were only present in *Sc* laboratory and bioethanol and some clinical strains. All flocculins except FLO5 protein and adhesins were present across all *Sb*, and we found that these have a larger number of repeats comparable to most of the *Sc* strains probably enabling adhesive properties to the organism.

The introgression of five genes, related to transporters, from *Z. bailii* ISA1307 to *Sb* was found to be present in similar fashion in *Sc* wine strains too. The taxonomic position of *Sb* was derived using 182 core proteins as the high genomic relatedness between *Sb* and *Sc* did not allow a few molecular markers to resolve the phylogeny. Taxonomically the probiotic yeast shares the clade with *Sc* UFMG A-905 and wine strains. In light of the genomic and taxonomic evidence we found that the probiotic yeast is closer to the wine strains as compared to industrial strains.

## Electronic supplementary material

Supplementary Information

Supplementary File I

Supplementary File II

Supplementary File III

Supplementary File IV

Supplementary File V

Supplementary File VI

Supplementary File VII

Supplementary File VIII

Supplementary File IX

Supplementary File X
