# Supplementary material for: Complete genome sequence and comparative genomics of the probiotic yeast Saccharomyces boulardii
Source: Sci Rep. 2017 Mar 23;7:371. doi: 10.1038/s41598-017-00414-2 (PMC5428479; doi:10.1038/s41598-017-00414-2)
Supplement: Supplementary file 1 — Supplementary Information [file 41598_2017_414_MOESM1_ESM.pdf]

TITLE

Complete genome sequence and comparative genomics of the probiotic yeast *Saccharomyces boulardii*.

Indu Khatri, Rajul Tomar, K. Ganesan, G.S. Prasad, and Srikrishna Subramanian[*]

Affiliations: CSIR-Institute of Microbial Technology, Chandigarh, India.

[*] Corresponding author: krishna@imtech.res.in

SUPPLEMENTARY MATERIALS

Isolation and purification of *Sb* genomic DNA

The lyophilized cells of *Sb* from Econorm, biocodex (Florastor) and unique28 and the cultures of *Sb-unisankyo* and *Sb-kirkman* maintained at MTCC, IMTECH were used as the source of the probiotic yeast (Table 1). Yeast cells were suspended in Milli-Q water, serially diluted, and plated on Yeast Malt (YM) agar (Difco) plates. The plates were incubated at 25°C for 48 hours. DNA isolation was performed using the ZR Fungal/Bacterial DNA miniprep kit (Zymogen) as per instructions in its user manual. After isolation, the genomic DNA was treated with RNase A (1µl of a ten µg/mL stock solution for 100µl of a solution containing DNA) and incubated at 37ºC for 30 minutes. Then, 1/10 volume of 3M sodium acetate (pH 5.2) and 2.5 volumes of absolute ethanol was added followed by incubation at -20ºC overnight and centrifugation at 14,000 rpm for 30 minutes at 4ºC. The supernatant was carefully discarded; the pellet was rinsed with 70% ethanol and centrifuged again at 14,000 rpm for 15 min at 4ºC. The ratio of OD at 260/280 nm was >1.8 as observed by NanoDropND-1000 spectrophotometer.

Internal Transcribed Spacer-Polymerase Chain Reaction (ITS-PCR)

ITS regions of all strains of *Sb* were amplified using ITS1 (TCCGTAGGTGAACCTGCGG) and ITS4 (TCCTCCGCTTATTGATATG) primers. Amplification was done using a mixture containing 1X Standard *Taq* Reaction Buffer, dNTPs (200 µM), BSA (0.3 µg/µL), template DNA (500 ng), 1.25 units/50 µl PCR *Taq* DNA Polymerase (Taq DNA Polymerase with Standard Taq Buffer; New England BioLabs) and forward and reverse primers (0.2 µM each). The cycling parameters used for amplification were: initial denaturation for 5 minutes at 95°C, followed by 30 cycles of 30 seconds at 95°C, 30 seconds at 50°C and 90 seconds at 72°C, with a final extension for 10 minutes at 72°C and cooling to 4°C. The amplified products were separated on 0.8% agarose gels, visualized and photographed on an Alpha Image Analyzer (Alpha Innotech Corporation, CA). The DNA from the amplified bands was eluted with QIAquick Gel Extraction Kit (Qiagen N.V.). The eluted DNA was further amplified for sequencing using Terminator Ready Reaction Mix (1µl), Sequencing Buffer (1µl; 5X; 200mM Tris-Cl, 5mM $MgCl_2$, pH 9.0), PCR amplified DNA (35ng), primer (3.2 pmol) and Milli-Q water to make up the volume to 10µl. PCR cycling conditions were: initial denaturation for 1 minute at 96°C, followed by 24 cycles of 10 seconds at 96°C, 5 seconds at 50°C and 4 minutes at 60°C and cooling to 4°C. The final PCR product was sequenced on a Sanger sequencer and the resulting ITS sequence was compared against all available ITS sequences in the NCBI database.

Microsatellite fingerprinting

Microsatellite fingerprinting based on the Post-Meiotic Segregation (PMS) marker PMS2 [1] was performed on all 5 strains of *Sb*, 2 strains of *Sc* MTCC 1766 and MTCC 4000 available in MTCC, IMTECH, India and one strain of *Kluyveromyces lactis* MTCC 458 from MTTC to discriminate *Sb* strains from other yeasts. Amplification was achieved using a mixture containing 1X Standard *Taq* Reaction Buffer, dNTPs (200 µM), BSA (0.3 µg/µL), template DNA (500 ng), 1.25 units/50 µl PCR *Taq* DNA Polymerase (Taq DNA Polymerase with Standard *Taq* Buffer; New England BioLabs) and 0.2 µM of forward and reverse primers (PMS2: GACGACGACGACGAC). The following cycling parameters were used for microsatellite fingerprinting: initial denaturation for 5 minutes at 94°C, followed by 40 cycles of 1 minute at 94°C, 2 minutes at 45°C and 3 minutes at 72°C, with a final extension for 10 minutes at 72°C and cooling to 4°C. The amplified products were separated on 1.2% agarose gels, visualized and photographed on an Alpha Image Analyzer (Alpha Innotech Corporation, CA). The PMS2 marker microsatellite fingerprinting verified the *Sb* strains similar to each other and different to other *Sc* strains and other yeasts (**Supplementary Figure 6).**

Sporulation phenotype

Also, yeast cells were streaked on YPD agar plates and incubated at 25°C for 48 hours. These cells were further sub-cultured onto GNA pre-sporulation plates (5% D-glucose, 3% Difco nutrient broth, 1% Difco yeast extract and 2% Bacto agar) at 30°C for 24 hours. A single colony was then transferred to sporulation broth (1% Potassium Acetate and 0.005% Zinc Acetate) and incubated for 3-5 days at different temperatures (25°C, 30°C and 37°C). Plates were visualized under OLYMPUS BX51 phase contrast microscope to trace the presence of spores. None of the plates exhibit the sporulation tetrad or spores on them.

Sb growth on Non-Fermentable Carbon source

Sb strains and *Sc* MTCC36^T were grown on YPG media along with glycerol (a non-fermentable carbon source). The media composition was (1% Bacto-yeast extract 10 g, 2% Bacto-peptone 20, 3% (v/v) Glycerol 30 ml, 2% Bacto-agar 20 g and Distilled water 970 ml). The plate was incubated at 25°C for 48 hours. The *Sb* strains grow on the non-fermenatble carbon source **(Supplementary Figure 3).** The growth of *Sb* strains was also observed in other non-fermentable carbon source as (Rhamnose, Ethanol, Sucrose, Fructose, Lactose, Dextrose and Acetate).

Genome Sequencing

PacBio P6C4: *Sb-biocodex* and *Sb-unique28* were sequenced using Pacbio P6C4 chemistry. SMRTbell libraries were created using the 'Procedure and Checklist–20 kb Template Preparation Using BluePippin™ Size Selection System protocol. DNA samples are sheared and concentrated using AMPure magnetic beads and treated by ExoVII to remove single stranded ends. DNA Damage Repair and End Repair steps are followed by purification of DNA. Blunt ligation reactions are prepared, and SMRTbell templates are purified using AMPure magnetic beads. BluePippin Size Selection is performed to retain longer reads for sequencing. The Size-selected SMRTbell templates are annealed, and the polymerase is added for Sequencing. Nine SMRT Cells for *Sb-biocodex* and 8 SMRT Cells for *Sb-unique28* were run on the PacBio RS II system using P6C4 chemistry and an 180-minute data collection mode at Genome Quebec Centre, McGill University, Canada.

Illumina HiSeq PE: *Sb-EDRL* (Econorm-Dr. Reddy's Laboratories), *Sb-unisankyo*, *Sb-kirkman* and *Sb-biocodex* were sequenced using the Illumina HiSeq PE platform. The library preparation was carried out according to the Tru Seq DNA sample preparation protocol (Illumina, Inc., San Diego, CA) at C-CAMP, Bangalore, India. One µg of bacterial DNA was sheared to an average length of 300 to 400 bp. End repair, A-tailing and adapter ligation (~120 base adapter) was performed according to paired-end DNA sample preparation kit (Illumina, Inc.). Size selection of adapter ligated DNA was done in a range of 400 to 550 bases for DNA library. The insert size was taken in a range of 280 to 430 bases for DNA library. PCR enrichment was performed for eight cycles, and the samples were validated on a Bioanalyzer. Libraries were sequenced in a Paired End 100 base run, using TruSeq PE Cluster Kit v3-cBot-HS for cluster generation on C-bot and TruSeq SBS Kit v3-HS (Catalog No.: PE-401-3001) for sequencing on the Illumina HiSeq 1000 platform according to recommended protocols.

Illumina HiSeq MP: *Sb-EDRL* and *Sb-kirkman* were sequenced using the Illumina HiSeq PE platform. The library preparation was carried out according to the Nextera Mate-Pair sample preparation protocol (Illumina, Inc., San Diego, CA) at C-CAMP, Bangalore, India. The Nextera Mate-Pair Tagment enzyme simultaneously fragments and attaches an adapter to the end of dsDNA. The tagmented molecules are then circularized, and the ends of the genomic DNA are linked by two copies of biotin junction adapter. These fragments are subsequently fragmented yielding smaller fragments of which the sub-fragments containing the original junction were enriched via biotin tag in the junction adapter. Further End Repair and A-Tailing are followed by addition of TruSeq DNA adapters, enabling amplification and sequencing.

Genome Assembly and Annotation:

***Sb* Assembly:** The probiotic *Sb* genomes marketed by different companies in lyophilized forms were used as a source for DNA isolation (**Supplementary Table 1**) and were sequenced by various sequencing technologies (**Supplementary Figure 1**). Single molecule, Real-Time

(SMRT) sequencing of *Sb-biocodex* and *Sb-unique28* using Pacbio RSII P6C4 chemistry was performed using nine and eight SMRT cells, respectively. These strains were assembled using Hierarchical Genome Assembly Process (HGAP) [2] v2.0 pipeline in SMRT Portal which resulted in 31 contigs for *Sb-biocodex* and 30 contigs for *Sb-unique28*. These contigs were subjected to the Basic Local Alignment Search Tool (BLAST) [3] against nucleotide collection (NT) database for any bacterial or plasmid contamination in the final assembly. No bacterial contamination was found, however two contigs from *Sb-biocodex* and one from *Sb-unique28* match to *Sc* strain YJM993 2 micron circle plasmid and was ultimately retrieved from the contigs. Also, two contigs from *Sb-unique28* hits to *Sc* S288C mitochondrion sequences, but could not be extracted completely. However, the reads of *Sb* when mapped to *Sc* S288C mitochondrion sequence as a reference we could obtain the complete mitochondrion sequence but with lesser coverage. The contigs with plasmids and mitochondrion hits were extracted separately, and the chromosomal assemblies without any plasmid and mitochondrion contigs were finalized with 29 contigs of *Sb-biocodex* (12 Mbp genome and N50 792,172 bp) and 27 contigs of *Sb-unique28* (12.1 Mb genome and N50 929,172 bp) **(Table 1)**. These contigs were mapped to the *Sc* S288C chromosomes using Mauve-Aligner, and the contigs were arranged according to [4] the respective chromosomes. The single contig mapped to one chromosome is finalized as a single chromosome. For *Sb-biocodex* 14 contigs were completely spanning the individual *Sc* S288C chromosomes except two which were further scaffolded using Illumina Paired-End (PE) (101 bp) data, and long PacBio reads in Microbial Genome Finishing (MGF) plugin of CLCbio wb8.0 (www.clcbio.com). For *Sb-biocodex*, all 16 chromosomes were obtained without any gaps. After finalizing chromosomes, we were still left with 12 contigs which could not be placed in any chromosomes and sum up to 0.2 Mbp of the total genome. These unplaced contigs were subjected to BLASTn against the finalized chromosomes. All these unplaced scaffolds found hits to the chromosomes. *Sb-unique28* likewise had 14 gapless contigs aligned to respective chromosomes except two chromosomes with two contigs spanning. Also, nine unplaced contigs were present in *Sb-unique28*, and all were matching to the chromosomes assembled **(Supplementary File I)**. The annotated ORFs of unplaced contigs in *Sb-biocodex* were mapped on to the ORFs obtained for the assembled chromosomes. We found most of these were > 90% identical to the chromosome ORFs except 4 ORFs which belongs to chromosome XIII. In *Sb-unique28* the unplaced ORFs were also >90% identical except one unique that belongs to Chromosome IX.

The repetitive regions, telomeres, at the end of chromatids, are present for protecting chromatids from deterioration or from combining with other neighbor chromosomes. The telomere sequences in SGD were subjected to BLASTn as a query against the *Sb-biocodex* and *Sb-unique28* genome sequence and the positions of telomere sequences were retrieved. Chromosomes assembled are complete regarding the presence of telomeres at the ends of the chromosomes and also the sizes of the chromosomes comparable to *Sc* S288C reference genome.

Ribosomal DNA Locus: The ribosomal DNA of *Sc* encoded by RDN1 Locus is approximately 1-2 Mb region where 100-200 copies of RDN1 locus were present on Chromosome XII [5]. Each repeat has 5S, 5.8S, 25S and 18S rRNA genes with internal (ITS1 and ITS2), external (5' ETS and 3' ETS) and non-transcribed (NTS1 and NTS2) spacers. The yeasts sequenced have two of the 100-200 rDNA repeats. We subjected this rDNA locus to *Sb-biocodex* genomes, and we could retrieve four complete copies of these rDNA locus double of what has been mentioned in SGD database. The locus was present on chromosome XIII at positions 467016-484308, 485179-502467, 448862-466147 and 430750-448000.

Quality assurance: The genomic DNA was purified from a commercially available lyophilized *Sb* (Econorm, Unique28, and Florastor) and maintained cultures of *Sb-kirkman* and *Sb-unisankyo* which were further confirmed by ITS sequencing. The ITS sequences from all the *Sb* strains were 100% identical to each other. The ITS sequence for all *Sb* was >99.8% identical to that of the *S. boulardii* 28S ribosomal RNA gene, partial sequence; internal transcribed spacer 2, 5.8S ribosomal RNA gene, and internal transcribed spacer 1, complete sequence; and 18S ribosomal RNA gene, partial sequence (AY428861.1). All the ITS sequences were submitted to NCBI.

Genome Annotation: Genome annotation for all the *Sb* genomes and *Sc* UFMG A-905 strain was performed through the MAKER, GeneMark, SNAP, and Augustus. We checked for the overlapping ORFs and the ORFs containing introns and mapped to the S288C genes. We found that Augustus had a larger number of predictions as compared to the other genome annotation software. The genes from *Sc* S288C genome were mapped on to the *Sb* predicted annotations and the accuracy of annotated regions were checked. The proteins which were reported to be absent in the manuscript were verified by mapping genes to the genome and further if any mutation in start codon has resulted in the unidentified ORF.

Adhesion proteins

Sb, binds to the pathogenic bacteria, inhibiting their interaction with intestinal receptors and subsequently prevents their host invasion. A previous study reports 44 cell wall proteins deletion mutants in *Sc* BY4741 where 5 out of 44 mutants (YJL158C (CIS3), YKL096W-A (CWP2), YMR306W (FKS3), YKL163W (PIR3) and YGR279C (SCW4)) lost their adherence ability to pathogenic bacteria [6]. The occurrence of these proteins was studied in all strains of *Sb* and the presence and absence matrix in comparison to all *Sc* strains. All 44 cell-wall proteins AGA2 were present in all strains of *Sb* and 60 strains of *Sc* as YJM1083, YJM1129, YJM1133, YJM1190, YJM1202, YJM1244, YJM1273, YJM1304, YJM1311, YJM1326, YJM1332, YJM1336, YJM1338, YJM1342, YJM1355, YJM1383, YJM1385, YJM1389, YJM1400, YJM1402, YJM1415, YJM1417, YJM1419, YJM1433, YJM1434, YJM1443, YJM1444, YJM1447, YJM1450, YJM1479, YJM1527, YJM1549, YJM1573, YJM1574, YJM1592, YJM189, YJM193, YJM195, YJM270, YJM271, YJM320, YJM326, YJM428, YJM450,

YJM453, YJM456, YJM541, YJM555, YJM627, YJM682, YJM683, YJM969, YJM978, YJM981, YJM984, YJM987, YJM993, YJM996, S288C and UFMG A-905 (**Supplementary File VII**).

63-kDa Phosphatase

E. coli toxin is inhibited by a 63-kDa phosphatase of *Sb* (PHO8P) containing a peptide (HFIGSSRTRSSDSLVTDSAAGATAFACALKSYNGAIGV) of 38-amino acids, by dephosphorylation of toxin [7]. The *pho8* gene with the synteny is conserved across all strains of *Sb* and majority of *Sc* strains [8]. The protein was conserved among all the *S. cerevisiae* strains with >95% identity with conserved activation site (VTDSAAGAT) and conserved neighborhood (**Supplementary Figure 7**).

54-kDa serine protease

Membrane-bound 54 kDa serine protease of *Sb* hydrolyzes toxins A and B of *C. difficile* to protect against *C. difficile*-induced diarrhea and colitis [9]. To retrieve the serine protease from these *Sb* strains the proteins in the molecular weight range of 45-60 kDa (1000 proteins) were retrieved and subjected to BLASTp against the MEROPS database [10]. The hits (14-15 proteins) obtained to the family of serine protease was screened for their complete coverage in the peptidase unit region of MEROPS entries. Finally, 8-9 proteins that belonged to S08, S09, S10 and S33 families of serine protease were retrieved. S8 belongs to 'SB*'* superfamily (Subtilases) that are non-specific protease whereas S09, S10 and S33 belong to 'SC' superfamily (**Supplementary File IX**). S9 includes prolyl oligopeptidase, dipeptidyl-peptidase IV, acylaminoacyl-peptidase and glutamyl endopeptidase C; S10 includes carboxypeptidase Y, and S33 includes prolyl aminopeptidase. These proteins were subjected to BLASTp against NR database and their functional roles were predicted. Most of these proteins had enzymatic functions as an acetyltransferase, sterol acetylase, and ester hydrolase. Of four proteins, two belonged to subtilisin family (Subtilisin-like Protease III) and two to vacuolar carboxypeptidase Y family were retrieved finally similar to the hits obtained previously in *Sb* genome study [8].

The molecular weights of Carboxypeptidases identified in *Sb* were 57 kDa and 59 kDa whereas those belonged to Subtilisin family have 53 kDa and 55 KDa. The *Sb* subtilisin family protein with 55 kDa molecular weight is OSW3 peptidase which works as a molecular chaperone and is secreted for proper spore wall assembly [11]. The other subtilisin with 53 kDa protein has been annotated as a hypothetical protein in MEROPS database and as a putative precursor to the subtilisin-like protease III in SGD. 53 KDa Mol wt protein of subtilisin family owing to its serine protease activity that functions against fungal pathogens [12] make it the most putative 54 kDa protein.

Sugar utilization potential of *Sb*

Galactose metabolism: The conversion of galactose to glucose-6-phosphate is carried out by the enzymes galactose-mutarotase, galactokinase, galactose-1-phosphate uridyltransferase, UDP-galactose-4-epimerase, and phosphoglucomutase, which are constituents of the Leloir pathway [13]. Glucose-6-phosphate is further utilized via glycolysis. The synteny of galactose utilization genes was found to be conserved similar to the majority of the *Sc* strains; the results concordant to our previous study [8]. The folds and the active site residues were also conserved in Galactokinase (*gal1*), galactose-1-phosphate uridyltransferase (*gal7*) and UDP-galactose-4-epimerase (*gal10*) sequences of *Sb* with a comparison to *Sc* sequences (**Supplementary Figure 9 and Supplementary File X**). The *Sc* strain S288C is a *gal2* strain that harbors all genes for galactose utilization but cannot utilize galactose when oxygen is limited [14]. The recessive *gal2* and *imp1* allele exhibit such phenotype which could not be traced out by genomics study [15].

Palatinose metabolism: Palatinose uptake and metabolism are performed by isomaltase genes IMP1, IMP2 and IMP5 of which IMP1 and IMP2 have high affinity to palatinose [16]. IMA multigene families encoding 5 isomaltases (Ima1, Ima2, Ima3, Ima4, and Ima5) are 65% to 99% identical [17]. These five isoenzymes are oligo-α-1,6-glucosidases where Ima1p and Ima2p exhibit the maximum activity against palatinose followed by Ima5p [16]. Palatinose is not utilized by *Sb* as reported earlier but Ima1p and Ima5p were present in *Sb* and *Sc* strain UFMG A-905, but Ima2p, Ima3p, and Ima4p were absent as confirmed by mapping studies and BLASTn to complete genome of *Sb* (**Supplementary File X**). Though IMP2 is absent, IMP1 can individually metabolize the palatinose as per the affinity of IMP1 to palatinose [17].

REFERENCES

1 Mancera, E., Bourgon, R., Huber, W. & Steinmetz, L. M. Genome-wide survey of post-meiotic segregation during yeast recombination. *Genome biology* **12**, R36, doi:10.1186/gb-2011-12-4-r36 (2011).

2 Chin, C. S. *et al.* Nonhybrid, finished microbial genome assemblies from long-read SMRT sequencing data. *Nature methods* **10**, 563-569, doi:10.1038/nmeth.2474 (2013).

3 Altschul, S. F., Gish, W., Miller, W., Myers, E. W. & Lipman, D. J. Basic local alignment search tool. *Journal of molecular biology* **215**, 403-410, doi:10.1016/S0022-2836(05)80360-2 (1990).

4 Rissman, A. I. *et al.* Reordering contigs of draft genomes using the Mauve aligner. *Bioinformatics* **25**, 2071-2073, doi:10.1093/bioinformatics/btp356 (2009).

5 Venema, J. & Tollervey, D. Ribosome synthesis in Saccharomyces cerevisiae. *Annual review of genetics* **33**, 261-311, doi:10.1146/annurev.genet.33.1.261 (1999).

6 Tiago, F. C. *et al.* Adhesion to the yeast cell surface as a mechanism for trapping pathogenic bacteria by *Saccharomyces* probiotics. *Journal of medical microbiology* **61**, 1194-1207, doi:10.1099/jmm.0.042283-0 (2012).

7 Buts, J. P. *et al. Saccharomyces boulardii* produces in rat small intestine a novel protein phosphatase that inhibits *Escherichia coli* endotoxin by dephosphorylation. *Pediatric research* **60**, 24-29, doi:10.1203/01.pdr.0000220322.31940.29 (2006).

8 Khatri, I. *et al.* Gleaning evolutionary insights from the genome sequence of a probiotic yeast *Saccharomyces boulardii*. *Gut pathogens* **5**, 30, doi:10.1186/1757-4749-5-30 (2013).

9 Castagliuolo, I., Riegler, M. F., Valenick, L., LaMont, J. T. & Pothoulakis, C. *Saccharomyces boulardii* protease inhibits the effects of *Clostridium difficile* toxins A and B in human colonic mucosa. *Infection and immunity* **67**, 302-307 (1999).

10 Rawlings, N. D., Barrett, A. J. & Bateman, A. MEROPS: the database of proteolytic enzymes, their substrates and inhibitors. *Nucleic acids research* **40**, D343-350, doi:10.1093/nar/gkr987 (2012).

11 Suda, Y., Rodriguez, R. K., Coluccio, A. E. & Neiman, A. M. A screen for spore wall permeability mutants identifies a secreted protease required for proper spore wall assembly. *PloS one* **4**, e7184, doi:10.1371/journal.pone.0007184 (2009).

12 Yan, L. & Qian, Y. Cloning and heterologous expression of SS10, a subtilisin-like protease displaying antifungal activity from *Trichoderma harzianum*. *FEMS microbiology letters* **290**, 54-61, doi:10.1111/j.1574-6968.2008.01403.x (2009).

13 McCullough, M. J., Clemons, K. V., McCusker, J. H. & Stevens, D. A. Species identification and virulence attributes of *Saccharomyces boulardii* (nom. inval.). *Journal of clinical microbiology* **36**, 2613-2617 (1998).

14 Douglas, H. C. & Condie, F. The genetic control of galactose utilization in *Saccharomyces*. *Journal of bacteriology* **68**, 662-670 (1954).

15 Donnini, C., Lodi, T., Ferrero, I., Algeri, A. & Puglisi, P. P. Allelism of IMP1 and GAL2 genes of *Saccharomyces cerevisiae*. *Journal of bacteriology* **174**, 3411-3415 (1992).

16 Teste, M. A., Francois, J. M. & Parrou, J. L. Characterization of a new multigene family encoding isomaltases in the yeast *Saccharomyces cerevisiae*, the IMA family. *The Journal of biological chemistry* **285**, 26815-26824, doi:10.1074/jbc.M110.145946 (2010).

17 Deng, X. *et al.* Similarities and differences in the biochemical and enzymological properties of the four isomaltases from *Saccharomyces cerevisiae*. *FEBS open bio* **4**, 200-212, doi:10.1016/j.fob.2014.02.004 (2014).

Supplementary Figures

Sb strains	Company	NGS data				Genome		
		Illumina PE	Illumina MP	454	Pacbio	Contigs	N50 (bp)	Genome Size (bp)
EDRL	Dr. Reddy's Laboratories	101 bp	2K & 8K	SG		77	819,652	11.5
Biocodex	Biocodex	101 bp			9 cells	29	792,172	12.0
Unique 28	Unique Biotech				8 cells	27	929,172	12.1
Kirkman	Kirkman	101 bp	2K & 8K			115	621,720	11.7
Unisankyo	Unisankyo	101 bp				164	262,146	11.6

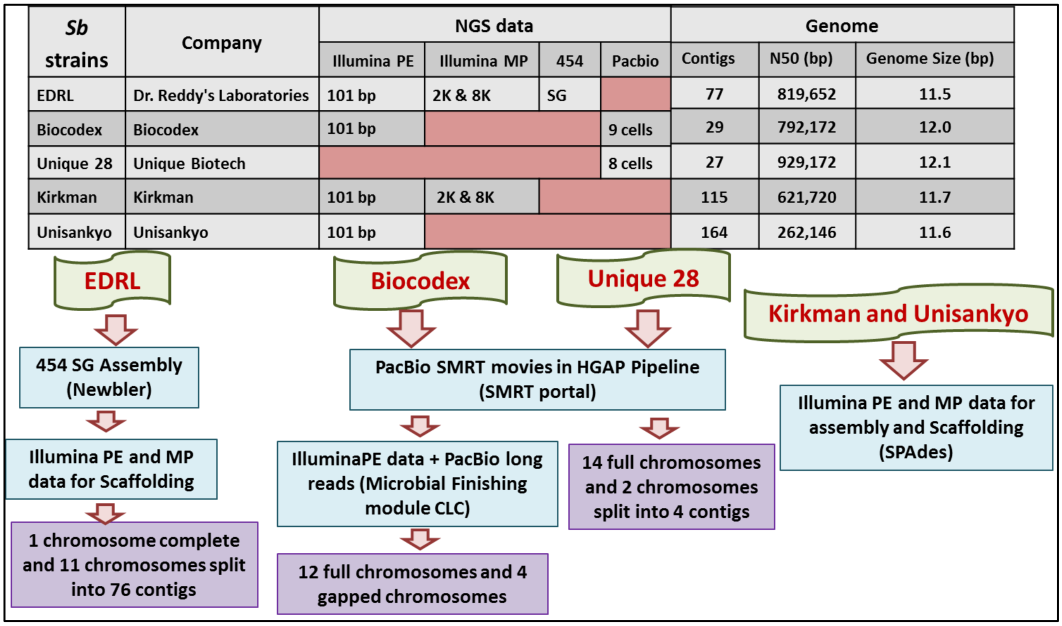

Supplementary Figure 1: Genome sequencing information, assembly statistics and the assembly methodology followed for all *Sb* strains.

```
Sb_biocodex_KP744576     41-TGCATTTTT-AAAA 135-GAC-AAACAG 200-AA-TAGA 5293-CATACCCT 5577-GAATGAATCAC
Sb_unique28_KP744573     41-TGCATTTTTTAAAA 136-GACCAAACAG 202-AAATAGA 5296-CATACCCT 5580-GAATGAATCAC
Sb_kirkman_KP744574      41-TGCATTTTT-AAAA 135-GAC-AAACAG 200-AAATAGA 5294-CATACCCT 5578-GAATGAATCAC
Sb_EDRL_KP744575         41-TGCATTTTT-AAAA 135-GAC-AAACAG 200-AAATAGA 5294-CATACCCT 5578-GAATGAATCAC
Sb_unisankyo_KP744577    41-TGCATTTTT-AAAA 135-GAC-AAACAG 200-AAATAGA 5294-CATACCCT 5578-GAATGAATCAC
Sc_YJM993_CP004528.1     41-TGCATTTTT-AAAA 135-GAC-AAACAG 200-AAATAGA 5294-CATGCCCT 5578-GAATAAATCAC
```

Supplementary Figure 2: The variations in 2 micron circle plasmid of *Sb* biocodex, *Sb* unique28, *Sb* krikman, *Sb* unisankyo and *Sc* YJM993 depicted in alignment. Substitutions have been marked in white color with pink background and insertion/deletions with cyan background.

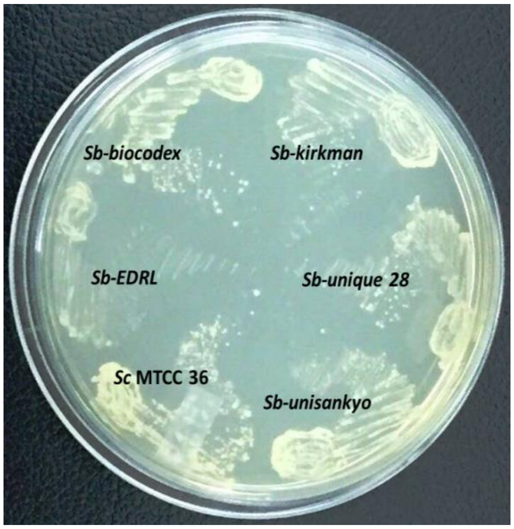

Supplementary Figure 3: The growth of *Sb* strains and Sc wild type strain MYCC36 on glycerol.

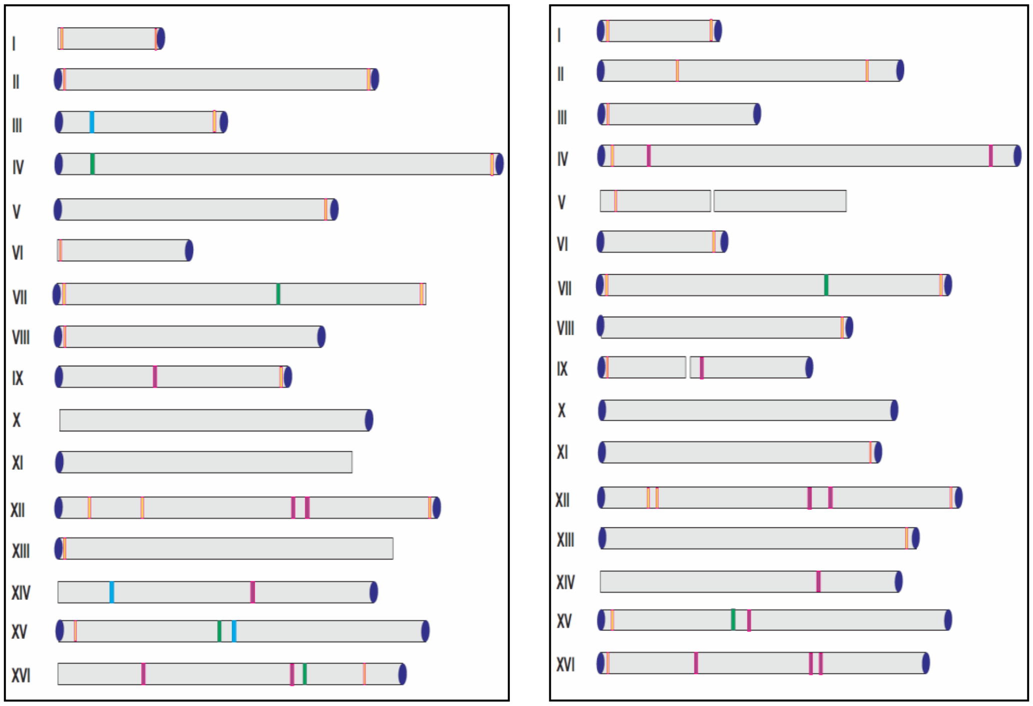

Supplementary Figure 4: Distribution of Ty elements and PAU proteins across chromosomes of *Sb-biocodex* (Panel A) and *Sb-unique28* (Panel B).

The chromosomes marked with blue ends have their telomeres sequenced. Here the locations of Ty elements (magenta), gag-co-pol genes (green) , gag genes (blue) and PAU proteins (yellow) has been depicted on the respective chromosomes at specified locations.

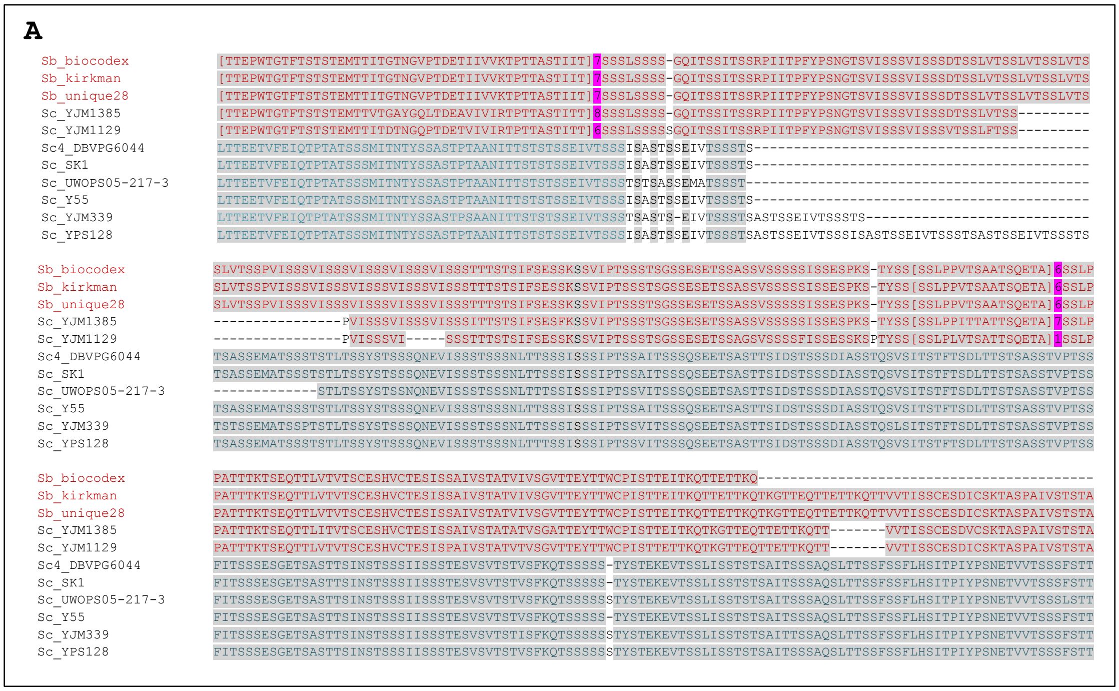

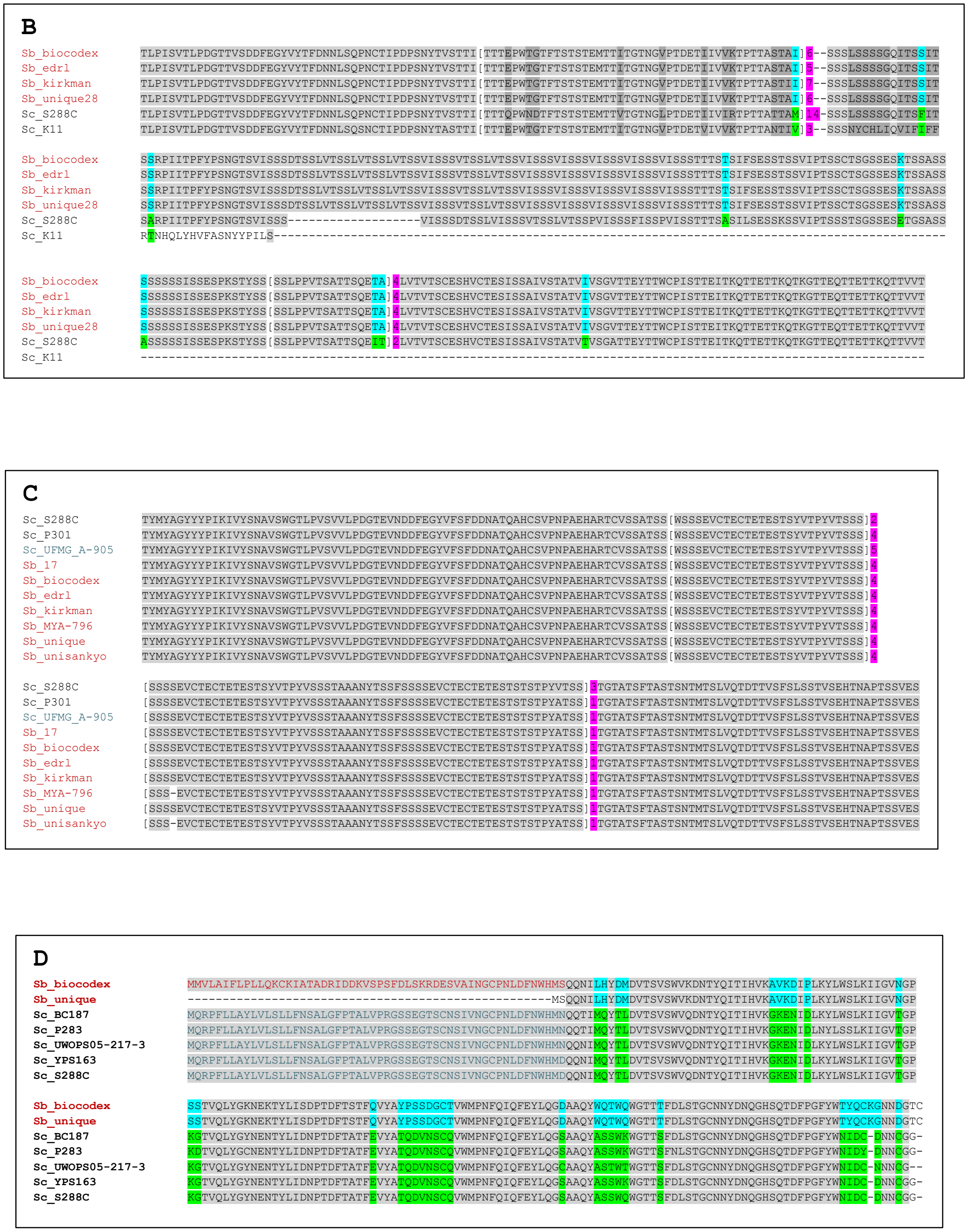

Supplementary Figure 5: The alignment of complete flocculin gene from all *Sb* and *Sc* strains. **A)** FLO1 **B)** FLO9 **C)** FO10 **D)** FLO11.

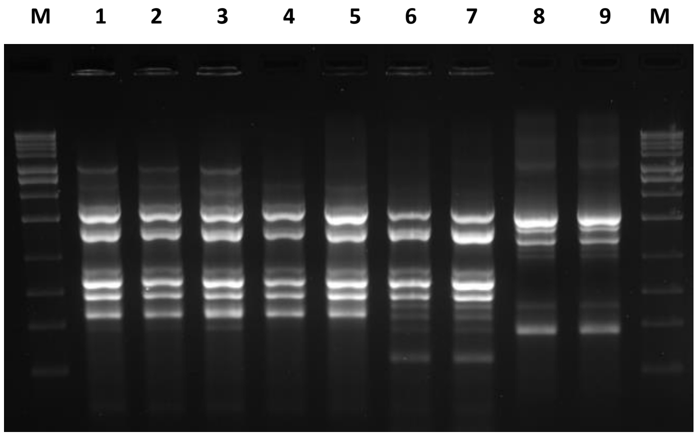

Supplementary Figure 6: Microsatellite fingerprinting of *Sb* with respect to other yeasts.
M- 1kb ladder; **1**- *Sb-kirkman*; 2- *Sb-EDRL*; 3- *Sb-unisankyo*; 4- *Sb-biocodex*; 5- *Sb* unique28; 6- *Sc* MTCC 1766; 7- *Sc* MTCC4000; 9- *Kluyveromyces lactis* MTCC 458.

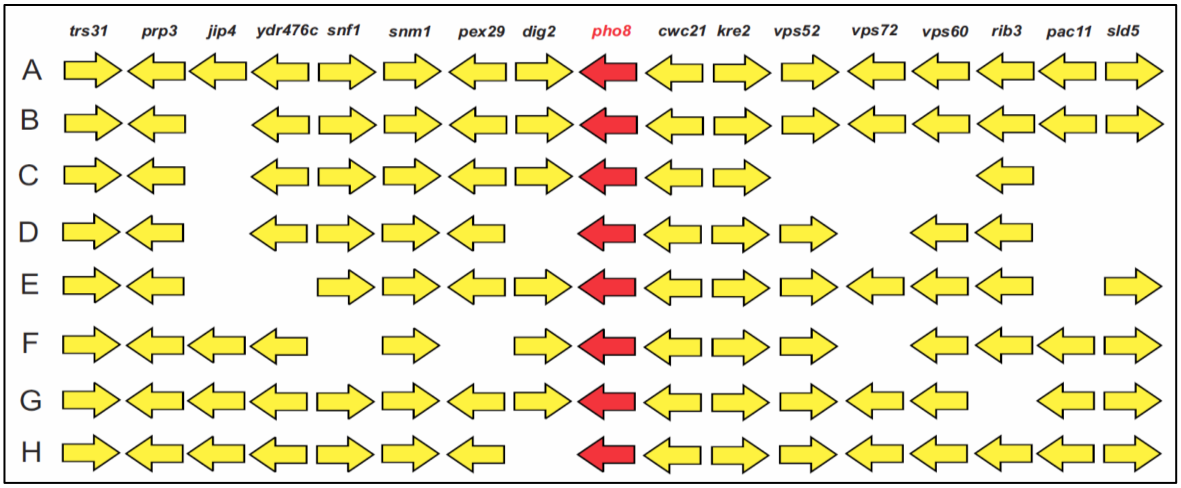

Supplementary Figure 7: Genome neighborhood of the 63 kDa phosphatase (PHO8) coding gene.

(A) *Sb* strains, and129 *Sc* strains **(B)** *Sc* strain EC9-8, P301 and R008; **(C)** *Sc strain* UC5; **(D)** *Sc strain* AWRI1631; **(E)** *Sc strain* YJM1447; **(F)** *Sc strain* CBS7960; **(G)** *Sc strain* PW5; **(H)** *Sc strain* P283. The genes mentioned are: *trs31* (YDR472W, Trapp subunit); *prp3* (YDR473C, Pre-mRNA processing); *jip4* (YDR475C, Jumonji domain interacting protein); YDR476C (Putative protein of unknown function; Green fluorescent protein (GFP)-fusion protein localizes to the endoplasmic reticulum); *snf1* (YDR477W, Sucrose non-fermenting); *snm1* (YDR478W, Suppressor of nuclear mitochondrial endoribonuclease 1); *pex2*9 (YDR479C, Peroxisomal integral membrane peroxin); *dig2* (YDR480W, Down-regulator of invasive growth); ***pho8p*** (YDR481C, Phosphate metabolism); *cwc21* (YDR482C, Complexed with Cef1p 1); *kre2* (YDR483W, Killer toxin resistant); vps52 (YDR484W, Vacuolar protein sorting); *vps72* (YDR485C, Vacuolar protein sorting 1); *vps60* (YDR486C, Vacuolar protein sorting); *rib3* (YDR487C, Riboflavin biosynthesis); *pac11* (YDR488C, Perish in the absence of CIN8 1) and *sld5* (YDR489W, Synthetic lethality with Dpb11-1).

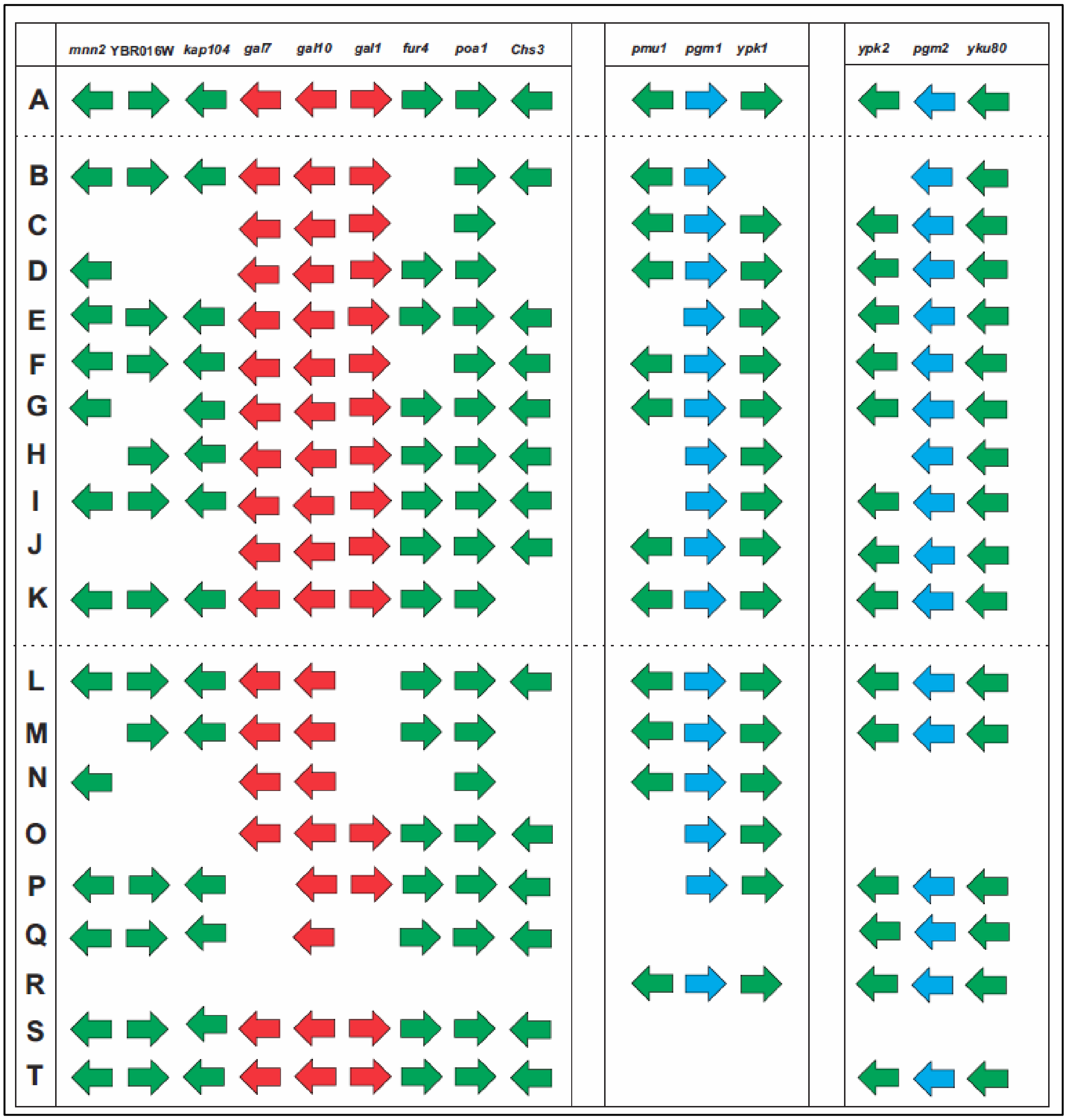

Supplementary Figure 8: Leloir pathway genes for galactose metabolism.

Genome context of genes involved in galactose metabolism. **(A)** *Sb* strains 112 strains of *Sc*; **(B)** *Sc* strain PW5 **(C)** *Sc* strain CBS7960; **(D)** *Sc strains* YJM269; **(E)** *Sc* strain JK9-3d; **(F)** *Sc* strains YJM1389 and YJM1592; **(G)** *Sc* strain YJM1447; **(H)** *Sc* strain P301; **(I)** *Sc* strains CEN.PK2-1Ca, W303 and R008 **(J)** *Sc* strains L1528 and UWOPS05-217-3; **(K)** *Sc* strain UC5; **(L)** *Sc* strain YJM1450; **(M)** *Sc* strain AWRI1631; **(N)** *Sc* strain CLIB215; **(O)** *Sc* strain EC9-8 AND YJM1250; **(P)** *Sc* strain P283; **(Q)** *Sc* strain YJM1342; **(R)** *Sc* strains R103, YJM1336, YJM1479, YJM244, YJM453 and CLIB324; **(S)** *Sc* strains FostersO and LalvinQA23; and **(T)** *Sc* strains YJM1399 and AWRI796. The genes shown are: *mnn2* (YBR015C, Mannosyltransferase); YBR016W; *kap104* (YBR017C, Karyopherin); *gal1* (YBR020W, Galactose metabolism); *gal7* (YBR018C,Galactose metabolism); *gal10* (YBR019C, Galactose metabolism); *fur4* (YBR021W, 5-Fluorouridine sensitivity); *poa1* (YBR022W, Phosphatase Of ADP-ribose 1′-phosphate); *chs3* (YBR023C, Chitin Synthase); *pmu1* (YKL128C, Phosphomutase); *pgm1* (YKL127W, Phosphoglucomutase); *ypk1* (YKL126W, Yeast protein kinase); *ypk2* (YMR104C, Yeast protein kinase); *pgm2* (YMR105C, Phosphoglucomutase); *yku80* (YMR106C, Yeast KU protein).

Supplementary Table1: The collection source of *Saccharomyces boulardii* strains.

***Sb* Company**	**Label**	**B. No.**	**Mfg. Date**	**Expiry Date**
**Dr. Reddy's Laboratories**	Econorm	1500	05-2012	04-2014
**Biocodex**	Florastor	1399	06-2011	11-2014
**Unique Biotech**	Unique 28	UBL/SAM/14/08/088	08-2014	07-2016

***Sb* Company**	**Culture Collection**
**Kirkman**	Culture maintained at MTCC since 2003
**Unisankyo**	Culture maintained at MTCC since 2003

Supplementary Files:

Supplementary File I: Genome statistics and assembly information of *Sb-biocodex* and *Sb-unique28* genomes.

Supplementary File II: Core and orthologous genes among all strains of *Sb*.

Supplementary File III: *Sb* and *Sc* genomes used for comparative analysis.

Supplementary File IV: Distribution of Sporulation genes, meiotic genes and mitotic genes across all *Sb* and *Sc* genomes.

Supplementary File V: Absent genes in Sb and Sc as compared to yeastmine database.

Supplementary File VI: Positions of Ty elements in all *Sb* strains.

Supplementary File VII: Distribution of flocculin genes and adhesion genes across all *Sb* and *Sc* genomes.

Supplementary File VIII: Average nucleotide identity calculated among all *Sb* and *Sc* genomes using BLASTn.

Supplementary File IX: The search space of 54-KDa protein in all *Sb* strains

Supplementary File X: Distribution of galactose and palatinose utilization genes in all *Sb* and *Sc* genomes.
